# Supplementary material for: On the inference of complex phylogenetic networks by Markov Chain Monte-Carlo
Source: PLoS Comput Biol. 2021 Sep 3;17(9):e1008380. doi: 10.1371/journal.pcbi.1008380 (PMC8445492; doi:10.1371/journal.pcbi.1008380)
Supplement: S1 Text — Fig A: Density probabilities for 5-tips networks, simulated with a prior corresponding to a birth hybridization process with parameters d = 10, r = 1/2 and τ0 = 0.1, using the SpeciesNetwork package [53]. The figure is obtained for 10,000 replicates. The means are given by the dashed vertical lines. Fig B: Density probabilities for 5-tips networks with at most two reticulations, simulated with a prior corresponding to a birth hybridization process with parameters d = 10, r = 1/2 and τ0 = 0.1, using the SpeciesNetwork package [53]. Figures are drawn for the 4,377 cases in 10,000 where the network had at most two reticulations. The means are given by the dashed vertical lines. Fig C: Density probabilities regarding the 5-tips network with a maximum of 3 reticulations, simulated under the birth hybridization process (d = 10, r = 1/2, τ0 = 0.1, 5,837 replicates), using the SpeciesNetwork package [53]. The means are given by the dashed vertical lines. Fig D: Estimated node heights of network B. 10,000 sites are considered and 2 lineages per species. Constant sites are included in the analysis, and the estimated heights are based on the 12 replicates (over 14 replicates) for which network B was recovered by SnappNet (criterion ESS > 200; θ ∼ Γ(1, 200), d∼E(0.1), r ∼ Beta(1, 1), τ0∼E(10) for the priors, number of reticulations bounded by 3 when exploring the network space). Heights are measured in units of expected number of mutations per site. True values are given by the dashed horizontal lines. The initials MRCA stand for “Most Recent Common Ancestor”. Fig E: Estimated population sizes θ for each branch of network B. Same framework as Figure D in S1 Text. True values are given by the dashed horizontal lines. The initials MRCA stand for “Most Recent Common Ancestor”. Fig F: Same framework as Figure E in S1 Text. Fig G: Estimated node heights of network C as a function of the number of sites. Same experiment as in Table 2 of the main manuscript: 1 lineage in species O, A [file pcbi.1008380.s001.pdf]

S1 Text. Supplementary material for the manuscript **"On the inference of complex phylogenetic networks by Markov Chain Monte-Carlo"**

August 6, 2021

Contents

1 A closer look at the rules **3**

1.1 Correctness of the rules for partial likelihoods. 3

1.2 About ranges 9

1.2.1 Observable number of lineages across the network 9

1.2.2 Ranges of the sums in Rules 2 and 4 10

2 Likelihood computation in detail **11**

3 Other computational complexity results **14**

3.1 Time complexity of the algorithm by Zhu et al. [1] 14

3.2 SnappNet's $\overline{K}$ and the level of the network 15

4 Newick representations **18**

5 `MCMCBiMarkers` commands **18**

6 Supplementary results for the simulation study **19**

7 Supplementary informations on rice real data **32**

8 **Additional experiments on SnappNet's MCMC sampler** **37**
8.1 Experiment with no data 37
8.1.1 Protocol 37
8.1.2 Results 38
8.2 Experiments on 10,000 simulated sites 44
8.2.1 Protocol 44
8.2.2 Results for network A 45
8.2.3 Results for network B 49
8.2.4 Operator acceptance rates 56

1 A closer look at the rules

Here, we first provide proofs of correctness for the rules to compute the partial likelihoods introduced in the main text (Sec. 1.1). Then we explain the rationale behind the ranges used for the summation terms in Rules 2 and 4 (Sec. 1.2).

1.1 Correctness of the rules for partial likelihoods.

Recall the definition of the partial likelihoods, which will be used in each of the proofs below:

$$\mathbf{F_x}\left(\mathbf{n_x};\mathbf{r_x}\right) = \mathbb{P}\left(R_{\mathbf{L(x)}} = \mathbf{r}_{\mathbf{L(x)}} \mid N_{\mathbf{x}} = \mathbf{n_x}, R_{\mathbf{x}} = \mathbf{r_x}\right) \times \mathbb{P}\left(N_{\mathbf{x}} = \mathbf{n_x}\right), \tag{1}$$

where $\mathbf{L}(\mathbf{x})$ is a vector of population interfaces (VPI) containing exactly once each leaf that descends from any element of $\mathbf{x}$.

We will also use the following equation (proven by Bryant et al. [2, 3] and based on [4]):

$$\mathbb{P}\left(R_{\underline{x}} = r_{\underline{x}} \mid N_{\underline{x}} = n_{\underline{x}}, N_{\overline{x}} = n_{\overline{x}}, R_{\overline{x}} = r_{\overline{x}}\right) = \frac{\exp(\mathbb{Q}_x t_x)_{(n_{\underline{x}},r_{\underline{x}});(n_{\overline{x}},r_{\overline{x}})}}{\mathbb{P}(N_{\overline{x}} = n_{\overline{x}} \mid N_{\underline{x}} = n_{\underline{x}})} \tag{2}$$

where $\mathbb{Q}_x = \left(q_{(n,r);(n',r')}\right)$ denotes the matrix with the following entries:

$$\begin{aligned}
q_{(n,r);(n,r-1)} &= (n-r+1)v & 0 < r \le n,\\
q_{(n,r);(n,r+1)} &= (r+1)u & 0 \le r < n,\\
q_{(n,r);(n-1,r)} &= \frac{(n-1-r)n}{\theta_x} & 0 \le r < n,\\
q_{(n,r);(n-1,r-1)} &= \frac{(r-1)n}{\theta_x} & 0 \le r \le n,\\
q_{(n,r);(n,r)} &= -\frac{n(n-1)}{\theta_x} - (n-r)v - ru & 0 \le r \le n,\\
q_{(n,r);(n',r')} &= 0 & \text{for all other entries.}
\end{aligned}$$

Finally, we note that many statements of conditional independence that we require in our proofs depend on the fact that the involved VPIs are incomparable.

Rule 0. *Let x be a branch incident to a leaf. Then,*

$$\mathbf{F}_{(\underline{x})}\left((n);(r)\right) = \mathbb{1}\{n = n_{\underline{x}}\} \times \mathbb{1}\{r = r_{\underline{x}}\}$$

Proof. Recall that the number of lineages sampled from species $\underline{x}$ is known and equal to $n_{\underline{x}}$. Then, applying definition (1) above with $\mathbf{x} = (\underline{x})$, we have:

$$\begin{aligned}
\mathbf{F}_{(\underline{x})}\left((n);(r)\right) &= \mathbb{P}\left(R_{\underline{x}} = r_{\underline{x}} \mid N_{\underline{x}} = n, R_{\underline{x}} = r\right) \times \mathbb{P}\left(N_{\underline{x}} = n\right)\\
&= \mathbb{1}\{r_{\underline{x}} = r\} \times \mathbb{1}\{n_{\underline{x}} = n\}.
\end{aligned}$$

□

Rule 1. *Let $\mathbf{x}, \underline{x}$ be a vector of incomparable population interfaces. Then,*

$$\mathbf{F}_{\mathbf{x},\overline{x}}\left(\mathbf{n}_{\mathbf{x}}, n_{\overline{x}}; \mathbf{r}_{\mathbf{x}}, r_{\overline{x}}\right) = \sum_{n=n_{\overline{x}}}^{m_x} \sum_{r=0}^{n} \mathbf{F}_{\mathbf{x},\underline{x}}\left(\mathbf{n}_{\mathbf{x}}, n; \mathbf{r}_{\mathbf{x}}, r\right) \exp(\mathbb{Q}_x t_x)_{(n,r);(n_{\overline{x}},r_{\overline{x}})}$$

Proof. First, note that, because $R_{\mathbf{L}(\mathbf{x},\overline{x})}$ is independent of $N_{\overline{x}}, R_{\overline{x}}$, when given $N_{\underline{x}}, R_{\underline{x}}$, and because $\mathbf{L}(\mathbf{x},\overline{x}) = \mathbf{L}(\mathbf{x},\underline{x})$:

$$\begin{aligned}
&\mathbb{P}\left(R_{\mathbf{L}(\mathbf{x},\overline{x})} = r_{\mathbf{L}(\mathbf{x},\overline{x})} \mid N_{\mathbf{x}} = \mathbf{n}_{\mathbf{x}}, R_{\mathbf{x}} = \mathbf{r}_{\mathbf{x}}, N_{\underline{x}} = n, R_{\underline{x}} = r, N_{\overline{x}} = n_{\overline{x}}, R_{\overline{x}} = r_{\overline{x}}\right) \\
&= \mathbb{P}\left(R_{\mathbf{L}(\mathbf{x},\underline{x})} = r_{\mathbf{L}(\mathbf{x},\underline{x})} \mid N_{\mathbf{x}} = \mathbf{n}_{\mathbf{x}}, R_{\mathbf{x}} = \mathbf{r}_{\mathbf{x}}, N_{\underline{x}} = n, R_{\underline{x}} = r\right)
\end{aligned}$$

Writing down the definition of $\mathbf{F}_{\mathbf{x},\overline{x}}$, then summing over all possible values of $N_{\underline{x}}$ and $R_{\underline{x}}$, and then using the identity above, we obtain:

$$\begin{aligned}
&\mathbf{F}_{\mathbf{x},\overline{x}}\left(\mathbf{n}_{\mathbf{x}}, n_{\overline{x}}; \mathbf{r}_{\mathbf{x}}, r_{\overline{x}}\right) \\
&= \sum_{n=n_{\overline{x}}}^{m_x} \sum_{r=0}^{n} \mathbb{P}\left(R_{\mathbf{L}(\mathbf{x},\underline{x})} = r_{\mathbf{L}(\mathbf{x},\underline{x})} \mid N_{\mathbf{x}} = \mathbf{n}_{\mathbf{x}}, R_{\mathbf{x}} = \mathbf{r}_{\mathbf{x}}, N_{\underline{x}} = n, R_{\underline{x}} = r\right) \\
&\times \mathbb{P}\left(N_{\underline{x}} = n, R_{\underline{x}} = r \mid N_{\mathbf{x}} = \mathbf{n}_{\mathbf{x}}, R_{\mathbf{x}} = \mathbf{r}_{\mathbf{x}}, N_{\overline{x}} = n_{\overline{x}}, R_{\overline{x}} = r_{\overline{x}}\right) \\
&\times \mathbb{P}\left(N_{\mathbf{x}} = \mathbf{n}_{\mathbf{x}}, N_{\overline{x}} = n_{\overline{x}}\right)
\end{aligned}$$

Moreover,

$$\begin{aligned}
&\mathbb{P}\left(N_{\underline{x}} = n, R_{\underline{x}} = r \mid N_{\mathbf{x}} = \mathbf{n}_{\mathbf{x}}, R_{\mathbf{x}} = \mathbf{r}_{\mathbf{x}}, N_{\overline{x}} = n_{\overline{x}}, R_{\overline{x}} = r_{\overline{x}}\right) \\
&= \mathbb{P}\left(R_{\underline{x}} = r \mid N_{\underline{x}} = n, N_{\overline{x}} = n_{\overline{x}}, R_{\overline{x}} = r_{\overline{x}}\right) \times \mathbb{P}\left(N_{\underline{x}} = n \mid N_{\mathbf{x}} = \mathbf{n}_{\mathbf{x}}, N_{\overline{x}} = n_{\overline{x}}\right),
\end{aligned}$$

where we have used that $R_{\underline{x}}$ is independent of $N_{\mathbf{x}}$ and $R_{\mathbf{x}}$, when given $N_{\underline{x}}, N_{\overline{x}}, R_{\overline{x}}$.

We then have:

$$\begin{aligned}
&\mathbf{F}_{\mathbf{x},\overline{x}}\left(\mathbf{n}_{\mathbf{x}}, n_{\overline{x}}; \mathbf{r}_{\mathbf{x}}, r_{\overline{x}}\right) \\
&= \sum_{n=n_{\overline{x}}}^{m_x} \sum_{r=0}^{n} \mathbb{P}\left(R_{\mathbf{L}(\mathbf{x},\underline{x})} = r_{\mathbf{L}(\mathbf{x},\underline{x})} \mid N_{\mathbf{x}} = \mathbf{n}_{\mathbf{x}}, R_{\mathbf{x}} = \mathbf{r}_{\mathbf{x}}, N_{\underline{x}} = n, R_{\underline{x}} = r\right) \\
&\times \mathbb{P}\left(R_{\underline{x}} = r \mid N_{\underline{x}} = n, N_{\overline{x}} = n_{\overline{x}}, R_{\overline{x}} = r_{\overline{x}}\right) \times \mathbb{P}(N_{\underline{x}} = n, N_{\mathbf{x}} = \mathbf{n}_{\mathbf{x}}, N_{\overline{x}} = n_{\overline{x}})
\end{aligned}$$

Using the fact that $N_{\overline{x}}$ is independent of $N_{\mathbf{x}}$, when given $N_{\underline{x}}$, the last term in the product can be rewritten as follows:

$$\mathbb{P}(N_{\underline{x}} = n, N_{\mathbf{x}} = \mathbf{n}_{\mathbf{x}}, N_{\overline{x}} = n_{\overline{x}}) = \mathbb{P}(N_{\overline{x}} = n_{\overline{x}} \mid N_{\underline{x}} = n) \times \mathbb{P}(N_{\underline{x}} = n, N_{\mathbf{x}} = \mathbf{n}_{\mathbf{x}})$$

Using Equation (2), we finally obtain:

$$\begin{aligned}
&\mathbf{F}_{\mathbf{x},\overline{x}}\left(\mathbf{n}_{\mathbf{x}}, n_{\overline{x}}; \mathbf{r}_{\mathbf{x}}, r_{\overline{x}}\right) \\
&= \sum_{n=n_{\overline{x}}}^{m_x} \sum_{r=0}^{n} \mathbb{P}\left(R_{\mathbf{L}(\mathbf{x},\underline{x})} = r_{\mathbf{L}(\mathbf{x},\underline{x})} \mid N_{\mathbf{x}} = \mathbf{n}_{\mathbf{x}}, R_{\mathbf{x}} = \mathbf{r}_{\mathbf{x}}, N_{\underline{x}} = n, R_{\underline{x}} = r\right) \\
&\times \mathbb{P}(N_{\underline{x}} = n, N_{\mathbf{x}} = \mathbf{n}_{\mathbf{x}}) \times \exp(\mathbb{Q}_x t_x)_{(n,r);(n_{\overline{x}}, r_{\overline{x}})} \\
&= \sum_{n=n_{\overline{x}}}^{m_x} \sum_{r=0}^{n} \mathbf{F}_{\mathbf{x},\underline{x}}(\mathbf{n}_{\mathbf{x}}, n; \mathbf{r}_{\mathbf{x}}, r) \times \exp(\mathbb{Q}_x t_x)_{(n,r);(n_{\overline{x}}, r_{\overline{x}})}
\end{aligned}$$

□

In the following proofs, to make the mathematics more readable, we denote each event $A = a$ inside a probability simply as a, whenever the left-hand side of $A = a$ is unambiguously determined by the right-hand side. For example:

$$\begin{aligned}
\mathbf{n}_{\mathbf{x}} &\quad \text{means} \quad N_{\mathbf{x}} = \mathbf{n}_{\mathbf{x}}, \\
\mathbf{r}_{\mathbf{x}} &\quad \text{means} \quad R_{\mathbf{x}} = \mathbf{r}_{\mathbf{x}}, \\
n_{\overline{x}} &\quad \text{means} \quad N_{\overline{x}} = n_{\overline{x}}, \\
r_{\overline{x}} &\quad \text{means} \quad R_{\overline{x}} = r_{\overline{x}}, \\
n_{\underline{x}} &\quad \text{means} \quad N_{\underline{x}} = n_{\underline{x}}, \\
r_{\underline{x}} &\quad \text{means} \quad R_{\underline{x}} = r_{\underline{x}}.
\end{aligned}$$

We will still write the full version in those cases where the left-hand side cannot be inferred from the right-hand side.

Rule 2. *Let $\mathbf{x}, \overline{x}$ and $\mathbf{y}, \overline{y}$ be two vectors of incomparable population interfaces, such that $\mathbf{L}(\mathbf{x}, \overline{x})$ and $\mathbf{L}(\mathbf{y}, \overline{y})$ have no leaf in common. Let x, y be the immediate descendants of branch z. Then,*

$$\begin{aligned}
&\mathbf{F}_{\mathbf{x},\mathbf{y},\underline{z}}\left(\mathbf{n}_{\mathbf{x}}, \mathbf{n}_{\mathbf{y}}, n_{\underline{z}}; \mathbf{r}_{\mathbf{x}}, \mathbf{r}_{\mathbf{y}}, r_{\underline{z}}\right) \\
&= \sum_{n_{\overline{x}}} \sum_{r_{\overline{x}}} \mathbf{F}_{\mathbf{x},\overline{x}}\left(\mathbf{n}_{\mathbf{x}}, n_{\overline{x}}; \mathbf{r}_{\mathbf{x}}, r_{\overline{x}}\right) \mathbf{F}_{\mathbf{y},\overline{y}}\left(\mathbf{n}_{\mathbf{y}}, n_{\underline{z}} - n_{\overline{x}}; \mathbf{r}_{\mathbf{y}}, r_{\underline{z}} - r_{\overline{x}}\right) \binom{n_{\overline{x}}}{r_{\overline{x}}} \binom{n_{\underline{z}} - n_{\overline{x}}}{r_{\underline{z}} - r_{\overline{x}}} \binom{n_{\underline{z}}}{r_{\underline{z}}}^{-1}
\end{aligned}$$

The ranges of $n_{\overline{x}}$ and $r_{\overline{x}}$ in the summation terms are defined by
$\max(0, n_{\underline{z}} - m_y) \leq n_{\overline{x}} \leq \min(m_x, n_{\underline{z}})$ *and*
$\max(0, n_{\overline{x}} + r_{\underline{z}} - n_{\underline{z}}) \leq r_{\overline{x}} \leq \min(n_{\overline{x}}, r_{\underline{z}})$.

Proof. By definition,

$$\mathbf{F}_{\mathbf{x},\mathbf{y},\underline{z}}\left(\mathbf{n}_{\mathbf{x}}, \mathbf{n}_{\mathbf{y}}, n_{\underline{z}}; \mathbf{r}_{\mathbf{x}}, \mathbf{r}_{\mathbf{y}}, r_{\underline{z}}\right) = \mathbb{P}\left(\mathbf{r}_{\mathbf{L}(\mathbf{x},\mathbf{y},\underline{z})} \mid \mathbf{n}_{\mathbf{x}}, \mathbf{n}_{\mathbf{y}}, n_{\underline{z}}, \mathbf{r}_{\mathbf{x}}, \mathbf{r}_{\mathbf{y}}, r_{\underline{z}}\right) \times \mathbb{P}\left(\mathbf{n}_{\mathbf{x}}, \mathbf{n}_{\mathbf{y}}, n_{\underline{z}}\right)$$

We then sum over all possible realizations of $N_{\overline{x}}$ and $R_{\overline{x}}$, and obtain:

$$\begin{aligned}
&\mathbf{F}_{\mathbf{x},\mathbf{y},\underline{z}}\left(\mathbf{n_x},\mathbf{n_y},n_{\underline{z}};\mathbf{r_x},\mathbf{r_y},r_{\underline{z}}\right) = \\
&\sum_{n_{\overline{x}}}\sum_{r_{\overline{x}}}\mathbb{P}\left(\mathbf{r}_{\mathbf{L}(\mathbf{x},\mathbf{y},\underline{z})} \mid \mathbf{n_x},\mathbf{n_y},n_{\underline{z}},\mathbf{r_x},\mathbf{r_y},r_{\underline{z}},n_{\overline{x}},r_{\overline{x}}\right) \\
&\times\mathbb{P}\left(n_{\overline{x}},r_{\overline{x}} \mid \mathbf{n_x},\mathbf{n_y},n_{\underline{z}},\mathbf{r_x},\mathbf{r_y},r_{\underline{z}}\right)\times\mathbb{P}\left(\mathbf{n_x},\mathbf{n_y},n_{\underline{z}}\right),
\end{aligned}$$

where the ranges in the summation terms are the same as those in the statement.

Now recall that $\mathbf{L}(\mathbf{x},\overline{x})$ and $\mathbf{L}(\mathbf{y},\overline{y})$ are disjoint vectors and note that their concatenation is equivalent to $\mathbf{L}(\mathbf{x},\mathbf{y},\underline{z})$. This means that $\mathbf{r}_{\mathbf{L}(\mathbf{x},\mathbf{y},\underline{z})}$ can also be written as $\mathbf{r}_{\mathbf{L}(\mathbf{x},\overline{x})},\mathbf{r}_{\mathbf{L}(\mathbf{y},\overline{y})}$. Moreover, $N_{\underline{z}}=n_{\underline{z}}$ and $N_{\overline{x}}=n_{\overline{x}}$ implies $N_{\overline{y}}=n_{\underline{z}}-n_{\overline{x}}$, and similarly $R_{\underline{z}}=r_{\underline{z}}$ and $R_{\overline{x}}=r_{\overline{x}}$ implies $R_{\overline{y}}=r_{\underline{z}}-r_{\overline{x}}$. We can then write:

$$\begin{aligned}
&\mathbb{P}\left(\mathbf{r}_{\mathbf{L}(\mathbf{x},\mathbf{y},\underline{z})} \mid \mathbf{n_x},\mathbf{n_y},n_{\underline{z}},\mathbf{r_x},\mathbf{r_y},r_{\underline{z}},n_{\overline{x}},r_{\overline{x}}\right) \\
&=\mathbb{P}\left(\mathbf{r}_{\mathbf{L}(\mathbf{x},\overline{x})},\mathbf{r}_{\mathbf{L}(\mathbf{y},\overline{y})} \mid \mathbf{n_x},\mathbf{n_y},n_{\underline{z}},\mathbf{r_x},\mathbf{r_y},r_{\underline{z}},n_{\overline{x}},r_{\overline{x}},N_{\overline{y}}=n_{\underline{z}}-n_{\overline{x}},R_{\overline{y}}=r_{\underline{z}}-r_{\overline{x}}\right) \\
&=\mathbb{P}\left(\mathbf{r}_{\mathbf{L}(\mathbf{x},\overline{x})} \mid \mathbf{n_x},\mathbf{r_x},n_{\overline{x}},r_{\overline{x}}\right)\times\mathbb{P}\left(\mathbf{r}_{\mathbf{L}(\mathbf{y},\overline{y})} \mid \mathbf{n_y},\mathbf{r_y},N_{\overline{y}}=n_{\underline{z}}-n_{\overline{x}},R_{\overline{y}}=r_{\underline{z}}-r_{\overline{x}}\right).
\end{aligned}$$

In the last equality above, we used the fact that $R_{\mathbf{L}(\mathbf{x},\overline{x})}$ and $R_{\mathbf{L}(\mathbf{y},\overline{y})}$ are independent random variables, given $N_{\mathbf{x},\overline{x}},R_{\mathbf{x},\overline{x}}$ and $N_{\mathbf{y},\overline{y}},R_{\mathbf{y},\overline{y}}$, respectively.

Moreover,

$$\begin{aligned}
&\mathbb{P}\left(n_{\overline{x}},r_{\overline{x}} \mid \mathbf{n_x},\mathbf{n_y},n_{\underline{z}},\mathbf{r_x},\mathbf{r_y},r_{\underline{z}}\right) \\
=\;&\mathbb{P}\left(r_{\overline{x}} \mid n_{\overline{x}},\mathbf{n_x},\mathbf{n_y},n_{\underline{z}},\mathbf{r_x},\mathbf{r_y},r_{\underline{z}}\right)\times\mathbb{P}\left(n_{\overline{x}} \mid \mathbf{n_x},\mathbf{n_y},n_{\underline{z}},\mathbf{r_x},\mathbf{r_y},r_{\underline{z}}\right) \\
&=\;\mathbb{P}\left(r_{\overline{x}} \mid n_{\overline{x}},n_{\underline{z}},r_{\underline{z}}\right)\times\mathbb{P}\left(n_{\overline{x}} \mid \mathbf{n_x},\mathbf{n_y},n_{\underline{z}}\right),
\end{aligned}$$

where in the last equality we have used the fact that $R_{\overline{x}}$ is independent of $N_{\mathbf{x}},N_{\mathbf{y}},R_{\mathbf{x}},R_{\mathbf{y}}$, when given $N_{\overline{x}},N_{\underline{z}}$, $R_{\underline{z}}$, and the fact that $N_{\overline{x}}$ is independent of $R_{\mathbf{x}},R_{\mathbf{y}},R_{\underline{z}}$, when given $N_{\mathbf{x}},N_{\mathbf{y}},N_{\underline{z}}$.

Putting all this together, we get:

$$\begin{aligned}
&\mathbf{F}_{\mathbf{x},\mathbf{y},\underline{z}}\left(\mathbf{n_x},\mathbf{n_y},n_{\underline{z}};\mathbf{r_x},\mathbf{r_y},r_{\underline{z}}\right) = \\
&\sum_{n_{\overline{x}}}\sum_{r_{\overline{x}}}\mathbb{P}\left(\mathbf{r}_{\mathbf{L}(\mathbf{x},\overline{x})} \mid \mathbf{n_x},\mathbf{r_x},n_{\overline{x}},r_{\overline{x}}\right)\times\mathbb{P}\left(\mathbf{r}_{\mathbf{L}(\mathbf{y},\overline{y})} \mid \mathbf{n_y},\mathbf{r_y},N_{\overline{y}}=n_{\underline{z}}-n_{\overline{x}},R_{\overline{y}}=r_{\underline{z}}-r_{\overline{x}}\right) \\
&\times\mathbb{P}\left(r_{\overline{x}} \mid n_{\overline{x}},n_{\underline{z}},r_{\underline{z}}\right)\times\mathbb{P}\left(n_{\overline{x}},\mathbf{n_x},\mathbf{n_y},n_{\underline{z}}\right).
\end{aligned}$$

Now note that

$$\begin{aligned}
\mathbb{P}\left(n_{\overline{x}},\mathbf{n_x},\mathbf{n_y},n_{\underline{z}}\right)&=\mathbb{P}\left(\mathbf{n_x},\mathbf{n_y},n_{\overline{x}},N_{\overline{y}}=n_{\underline{z}}-n_{\overline{x}}\right) \\
&=\mathbb{P}\left(\mathbf{n_x},n_{\overline{x}}\right)\times\mathbb{P}\left(\mathbf{n_y},N_{\overline{y}}=n_{\underline{z}}-n_{\overline{x}}\right),
\end{aligned}$$

where the last equality is due to the independence between the lineages from $\mathbf{L}(\mathbf{x},\overline{x})$ and those from $\mathbf{L}(\mathbf{y},\overline{y})$.

Finally, $R_{\overline{x}}$, given $N_{\overline{x}} = n_{\overline{x}}, N_{\underline{z}} = n_{\underline{z}}, R_{\underline{z}} = r_{\underline{z}}$ follows a hypergeometric distribution:

$$\mathbb{P}\left(r_{\overline{x}} \mid n_{\overline{x}}, n_{\underline{z}}, r_{\underline{z}}\right) = \binom{n_{\overline{x}}}{r_{\overline{x}}}\binom{n_{\underline{z}} - n_{\overline{x}}}{r_{\underline{z}} - r_{\overline{x}}}\binom{n_{\underline{z}}}{r_{\underline{z}}}^{-1}, \tag{3}$$

which allows us to conclude:

$$\begin{aligned}
\mathbf{F}_{\mathbf{x},\mathbf{y},\underline{z}}\left(\mathbf{n_x}, \mathbf{n_y}, n_{\underline{z}}; \mathbf{r_x}, \mathbf{r_y}, r_{\underline{z}}\right) &= \sum_{n_{\overline{x}}}\sum_{r_{\overline{x}}} \mathbb{P}\left(\mathbf{r}_{\mathbf{L}(\mathbf{x},\overline{x})} \mid \mathbf{n_x}, \mathbf{r_x}, n_{\overline{x}}, r_{\overline{x}}\right) \times \mathbb{P}\left(\mathbf{n_x}, n_{\overline{x}}\right) \\
&\times \mathbb{P}\left(\mathbf{r}_{\mathbf{L}(\mathbf{y},\overline{y})} \mid \mathbf{n_y}, \mathbf{r_y}, N_{\overline{y}} = n_{\underline{z}} - n_{\overline{x}}, R_{\overline{y}} = r_{\underline{z}} - r_{\overline{x}}\right) \times \mathbb{P}\left(\mathbf{n_y}, N_{\overline{y}} = n_{\underline{z}} - n_{\overline{x}}\right) \\
&\times \binom{n_{\overline{x}}}{r_{\overline{x}}}\binom{n_{\underline{z}} - n_{\overline{x}}}{r_{\underline{z}} - r_{\overline{x}}}\binom{n_{\underline{z}}}{r_{\underline{z}}}^{-1} \\
&= \sum_{n_{\overline{x}}}\sum_{r_{\overline{x}}} \mathbf{F}_{\mathbf{x},\overline{x}}\left(\mathbf{n_x}, n_{\overline{x}}; \mathbf{r_x}, r_{\overline{x}}\right) \mathbf{F}_{\mathbf{y},\overline{y}}\left(\mathbf{n_y}, n_{\underline{z}} - n_{\overline{x}}; \mathbf{r_y}, r_{\underline{z}} - r_{\overline{x}}\right) \binom{n_{\overline{x}}}{r_{\overline{x}}}\binom{n_{\underline{z}} - n_{\overline{x}}}{r_{\underline{z}} - r_{\overline{x}}}\binom{n_{\underline{z}}}{r_{\underline{z}}}^{-1}.
\end{aligned}$$

□

Rule 3. *Let* $\mathbf{x}, \overline{x}$ *be a vector of incomparable population interfaces, such that branch* x*'s top node is a reticulation node. Let* y, z *be the branches immediately ancestral to* x*. Then,*

$$\mathbf{F}_{\mathbf{x},\underline{y},\underline{z}}\left(\mathbf{n_x}, n_{\underline{y}}, n_{\underline{z}}; \mathbf{r_x}, r_{\underline{y}}, r_{\underline{z}}\right) = \mathbf{F}_{\mathbf{x},\overline{x}}\left(\mathbf{n_x}, n_{\underline{y}} + n_{\underline{z}}; \mathbf{r_x}, r_{\underline{y}} + r_{\underline{z}}\right)\binom{n_{\underline{y}} + n_{\underline{z}}}{n_{\underline{y}}}\gamma_y^{n_{\underline{y}}} \cdot \gamma_z^{n_{\underline{z}}}$$

Proof. First note that

$$\mathbb{P}\left(\mathbf{r}_{\mathbf{L}(\mathbf{x},\underline{y},\underline{z})} \mid \mathbf{n_x}, n_{\underline{y}}, n_{\underline{z}}, \mathbf{r_x}, r_{\underline{y}}, r_{\underline{z}}\right) = \mathbb{P}\left(\mathbf{r}_{\mathbf{L}(\mathbf{x},\overline{x})} \mid \mathbf{n_x}, N_{\overline{x}} = n_{\underline{y}} + n_{\underline{z}}, \mathbf{r_x}, R_{\overline{x}} = r_{\underline{y}} + r_{\underline{z}}\right).$$

Then, using the definitions of $\mathbf{F}_{\mathbf{x},\underline{y},\underline{z}}$ and $\mathbf{F}_{\mathbf{x},\overline{x}}$:

$$\frac{\mathbf{F}_{\mathbf{x},\underline{y},\underline{z}}\left(\mathbf{n_x}, n_{\underline{y}}, n_{\underline{z}}; \mathbf{r_x}, r_{\underline{y}}, r_{\underline{z}}\right)}{\mathbf{F}_{\mathbf{x},\overline{x}}\left(\mathbf{n_x}, n_{\underline{y}} + n_{\underline{z}}; \mathbf{r_x}, r_{\underline{y}} + r_{\underline{z}}\right)} = \frac{\mathbb{P}\left(\mathbf{n_x}, n_{\underline{y}}, n_{\underline{z}}\right)}{\mathbb{P}\left(\mathbf{n_x}, N_{\overline{x}} = n_{\underline{y}} + n_{\underline{z}}\right)}$$

But

$$\frac{\mathbb{P}\left(\mathbf{n_x}, n_{\underline{y}}, n_{\underline{z}}\right)}{\mathbb{P}\left(\mathbf{n_x}, N_{\overline{x}} = n_{\underline{y}} + n_{\underline{z}}\right)} = \mathbb{P}\left(n_{\underline{y}}, n_{\underline{z}} \mid \mathbf{n_x}, N_{\overline{x}} = n_{\underline{y}} + n_{\underline{z}}\right) = \binom{n_{\underline{y}} + n_{\underline{z}}}{n_{\underline{y}}}\gamma_y^{n_{\underline{y}}} \cdot \gamma_z^{n_{\underline{z}}},$$

where the first equality applies the definition of conditional probability, and the second equality uses the fact that $N_{\underline{y}}$ and $N_{\underline{z}}$ are binomially distributed, when given $N_{\overline{x}}$. The Rule trivially follows. □

Rule 4. *Let* $\mathbf{z}, \overline{x}, \overline{y}$ *be a vector of incomparable population interfaces, and let* x, y *be immediate descendants of branch* z. *Then,*

$$\mathbf{F}_{\mathbf{z},\underline{z}}\left(\mathbf{n_z}, n_{\underline{z}}; \mathbf{r_z}, r_{\underline{z}}\right)$$
$$= \sum_{n_{\overline{x}}} \sum_{r_{\overline{x}}} \mathbf{F}_{\mathbf{z},\overline{x},\overline{y}}\left(\mathbf{n_z}, n_{\overline{x}}, n_{\underline{z}} - n_{\overline{x}}; \mathbf{r_z}, r_{\overline{x}}, r_{\underline{z}} - r_{\overline{x}}\right) \binom{n_{\overline{x}}}{r_{\overline{x}}} \binom{n_{\underline{z}} - n_{\overline{x}}}{r_{\underline{z}} - r_{\overline{x}}} \binom{n_{\underline{z}}}{r_{\underline{z}}}^{-1}$$

The ranges of $n_{\overline{x}}$ *and* $r_{\overline{x}}$ *in the sums are the same as those in Rule 2.*

Proof. Use the definition of $\mathbf{F}_{\mathbf{z},\underline{z}}$ and then sum over all possible realizations of $N_{\overline{x}}$ and $R_{\overline{x}}$:

$$\mathbf{F}_{\mathbf{z},\underline{z}}\left(\mathbf{n_z}, n_{\underline{z}}; \mathbf{r_z}, r_{\underline{z}}\right) = \mathbb{P}\left(\mathbf{r}_{\mathbf{L}(\mathbf{z},\underline{z})} \mid \mathbf{n_z}, n_{\underline{z}}, \mathbf{r_z}, r_{\underline{z}}\right) \times \mathbb{P}\left(\mathbf{n_z}, n_{\underline{z}}\right) =$$
$$\sum_{n_{\overline{x}}} \sum_{r_{\overline{x}}} \mathbb{P}\left(\mathbf{r}_{\mathbf{L}(\mathbf{z},\underline{z})} \mid \mathbf{n_z}, n_{\underline{z}}, \mathbf{r_z}, r_{\underline{z}}, n_{\overline{x}}, r_{\overline{x}}\right) \times \mathbb{P}(n_{\overline{x}}, r_{\overline{x}} \mid \mathbf{n_z}, n_{\underline{z}}, \mathbf{r_z}, r_{\underline{z}}) \times \mathbb{P}(\mathbf{n_z}, n_{\underline{z}})$$

Now note that $\mathbf{L}(\mathbf{z}, \underline{z}) = \mathbf{L}(\mathbf{z}, \overline{x}, \overline{y})$, and that

$$\begin{aligned} &N_{\underline{z}} = n_{\underline{z}}, R_{\underline{z}} = r_{\underline{z}}, N_{\overline{x}} = n_{\overline{x}}, R_{\overline{x}} = r_{\overline{x}} \quad \text{if and only if} \\ &N_{\overline{x}} = n_{\overline{x}}, R_{\overline{x}} = r_{\overline{x}}, N_{\overline{y}} = n_{\underline{z}} - n_{\overline{x}}, R_{\overline{y}} = n_{\underline{z}} - n_{\overline{x}}, \end{aligned}$$

meaning that

$$\begin{aligned} &\mathbb{P}\left(\mathbf{r}_{\mathbf{L}(\mathbf{z},\underline{z})} \mid \mathbf{n_z}, n_{\underline{z}}, \mathbf{r_z}, r_{\underline{z}}, n_{\overline{x}}, r_{\overline{x}}\right) \\ &= \mathbb{P}\left(\mathbf{r}_{\mathbf{L}(\mathbf{z},\overline{x},\overline{y})} \mid \mathbf{n_z}, \mathbf{r_z}, n_{\overline{x}}, r_{\overline{x}}, N_{\overline{y}} = n_{\underline{z}} - n_{\overline{x}}, R_{\overline{y}} = n_{\underline{z}} - n_{\overline{x}}\right). \end{aligned}$$

Moreover,

$$\begin{aligned} &\mathbb{P}(n_{\overline{x}}, r_{\overline{x}} \mid \mathbf{n_z}, n_{\underline{z}}, \mathbf{r_z}, r_{\underline{z}}) \\ &= \mathbb{P}(r_{\overline{x}} \mid n_{\overline{x}}, \mathbf{n_z}, n_{\underline{z}}, \mathbf{r_z}, r_{\underline{z}}) \times \mathbb{P}(n_{\overline{x}} \mid \mathbf{n_z}, n_{\underline{z}}, \mathbf{r_z}, r_{\underline{z}}) \\ &= \mathbb{P}(r_{\overline{x}} \mid n_{\overline{x}}, n_{\underline{z}}, r_{\underline{z}}) \qquad \times \mathbb{P}(n_{\overline{x}} \mid \mathbf{n_z}, n_{\underline{z}}), \end{aligned}$$

where in the last equality we have used that $r_{\overline{x}}$ is independent of $\mathbf{n_z}, \mathbf{r_z}$, when given $n_{\overline{x}}, n_{\underline{z}}, r_{\underline{z}}$, and the fact that $n_{\overline{x}}$ is independent of $\mathbf{r_z}, r_{\underline{z}}$, when given $n_{\underline{z}}$.

Now use again Equation (3) to express $\mathbb{P}(r_{\overline{x}} \mid n_{\overline{x}}, n_{\underline{z}}, r_{\underline{z}})$ and conclude:

$$\begin{aligned} &\mathbf{F}_{\mathbf{z},\underline{z}}\left(\mathbf{n_z}, n_{\underline{z}}; \mathbf{r_z}, r_{\underline{z}}\right) \\ &= \sum_{n_{\overline{x}}} \sum_{r_{\overline{x}}} \mathbb{P}\left(\mathbf{r}_{\mathbf{L}(\mathbf{z},\overline{x},\overline{y})} \mid \mathbf{n_z}, \mathbf{r_z}, n_{\overline{x}}, r_{\overline{x}}, N_{\overline{y}} = n_{\underline{z}} - n_{\overline{x}}, R_{\overline{y}} = n_{\underline{z}} - n_{\overline{x}}\right) \\ &\times \mathbb{P}(n_{\overline{x}} \mid \mathbf{n_z}, n_{\underline{z}}) \times \mathbb{P}(\mathbf{n_z}, n_{\underline{z}}) \times \binom{n_{\overline{x}}}{r_{\overline{x}}} \binom{n_{\underline{z}} - n_{\overline{x}}}{r_{\underline{z}} - r_{\overline{x}}} \binom{n_{\underline{z}}}{r_{\underline{z}}}^{-1} \\ &= \sum_{n_{\overline{x}}} \sum_{r_{\overline{x}}} \mathbf{F}_{\mathbf{z},\overline{x},\overline{y}}\left(\mathbf{n_z}, n_{\overline{x}}, n_{\underline{z}} - n_{\overline{x}}; \mathbf{r_z}, r_{\overline{x}}, r_{\underline{z}} - r_{\overline{x}}\right) \binom{n_{\overline{x}}}{r_{\overline{x}}} \binom{n_{\underline{z}} - n_{\overline{x}}}{r_{\underline{z}} - r_{\overline{x}}} \binom{n_{\underline{z}}}{r_{\underline{z}}}^{-1} \end{aligned}$$

□

1.2 About ranges

We start this section with a general discussion about the values that the random variables $N_{\underline{x}}, N_{\overline{x}}, R_{\underline{x}}, R_{\overline{x}}$ can take for any population interface in the network. As usual, we will use lower-case letters for their realizations, i.e. $n_{\underline{x}}, n_{\overline{x}}, r_{\underline{x}}, r_{\overline{x}}$. Our remarks will allow us to derive the ranges used in our rules as simple consequences of a few equations.

1.2.1 Observable number of lineages across the network

The number of lineages $n_{\underline{x}}, n_{\overline{x}}, r_{\underline{x}}, r_{\overline{x}}$ observed at any population interface in the network must satisfy a few simple and obvious constraints, which we list below:

- For any branch x, the number of lineages at the top of the branch is at least 1, unless there were no lineages at the bottom of the branch, and at most equal to the number of lineages at the bottom. That is,

$$\mathbb{1}\{n_{\underline{x}} > 0\} \leq n_{\overline{x}} \leq n_{\underline{x}} \tag{4}$$

- At any population interface, the number of red and green lineages cannot exceed the total number of lineages. That is, for any branch x:

$$0 \leq r_{\underline{x}} \leq n_{\underline{x}} \tag{5}$$

$$0 \leq r_{\overline{x}} \leq n_{\overline{x}} \tag{6}$$

- For any internal node u, the numbers of red and green lineages entering u are the same as the numbers of red and green lineages exiting u. That is, if u is a tree node with ingoing branch z and outgoing branches x, y:

$$n_{\underline{z}} = n_{\overline{x}} + n_{\overline{y}} \tag{7}$$

$$r_{\underline{z}} = r_{\overline{x}} + r_{\overline{y}} \tag{8}$$

  (Note that these two equations also imply that the numbers of green lineages entering and exiting u are the same.)

  If u is a reticulation with ingoing branches x, y and outgoing branch z:

$$n_{\overline{z}} = n_{\underline{x}} + n_{\underline{y}} \tag{9}$$

$$r_{\overline{z}} = r_{\underline{x}} + r_{\underline{y}} \tag{10}$$

- A simple consequence of Equations (4), (7) and (9) is that the number of lineages in any branch x cannot exceed the total number of lineages at the leaves that descend from x, that is:

$$n_{\underline{x}}, n_{\overline{x}} \leq m_x \tag{11}$$

  (This can easily be proven by induction on the height of x.)

Constraints (4)-(10) above are not only necessary, but also sufficient to describe all possible values of $n_{\underline{x}}, n_{\overline{x}}, r_{\underline{x}}, r_{\overline{x}}$ across the network. In theory they could be used to infer the precise ranges for these variables, starting from the leaves and moving up the network.

In practice, however, this is unnecessary. SnappNet only ensures that for any population interface $\underline{x}$ or $\overline{x}$, the following two equations are satisfied:

$$0 \leq r_{\underline{x}} \leq n_{\underline{x}} \leq m_x \tag{12}$$

$$0 \leq r_{\overline{x}} \leq n_{\overline{x}} \leq m_x \tag{13}$$

These equations also specify the ranges for which $\mathbf{F_x}(\mathbf{n_x}; \mathbf{r_x})$ is defined and stored in memory.

Note that equations (12) and (13) permit a few more values for the n arguments than are actually possible. For example $n_{\underline{x}}$ is allowed to be 0, even when this is not possible (e.g. when x lies on all paths from a leaf with sampled individuals to the root). Whenever this occurs, the probability term within $\mathbf{F_x}(\mathbf{n_x}; \mathbf{r_x})$ equals 0. As a result, the partial likelihood itself is 0 and does not contribute to the calculation of any partial likelihood higher up in the network.

1.2.2 Ranges of the sums in Rules 2 and 4

It is now easy to justify the ranges in the sums in Rules 2 and 4. Recall that both these rules describe the behavior of the algorithm when traversing a tree node with ingoing branch z and outgoing branches x, y. Also recall that these rules sum over the possible values for $n_{\overline{x}}$ and $r_{\overline{x}}$. Note that, because conservation constraints (7) and (8) must hold here, these values also determine the values of $n_{\overline{y}} = n_{\underline{z}} - n_{\overline{x}}$ and $r_{\overline{y}} = r_{\underline{z}} - r_{\overline{x}}$.

Let's first consider the range for $n_{\overline{x}}$. By applying constraint (13) to $n_{\overline{x}}$ and then $n_{\overline{y}}$, we must ensure:

$$0 \leq n_{\overline{x}} \leq m_x$$

$$0 \leq n_{\underline{z}} - n_{\overline{x}} \leq m_y$$

The second equation is equivalent to $n_{\underline{z}} - m_y \leq n_{\overline{x}} \leq n_{\underline{z}}$ and therefore we get:

$$\max(0, n_{\underline{z}} - m_y) \leq n_{\overline{x}} \leq \min(m_x, n_{\underline{z}})$$

As for $r_{\overline{x}}$, by applying constraint (13) to $r_{\overline{x}}$ and then $r_{\overline{y}}$, we must ensure:

$$0 \leq r_{\overline{x}} \leq n_{\overline{x}}$$

$$0 \leq r_{\underline{z}} - r_{\overline{x}} \leq n_{\underline{z}} - n_{\overline{x}}$$

The second equation is equivalent to $n_{\overline{x}} + r_{\underline{z}} - n_{\underline{z}} \leq r_{\overline{x}} \leq r_{\underline{z}}$ and therefore we get:

$$\max(0, n_{\overline{x}} + r_{\underline{z}} - n_{\underline{z}}) \leq r_{\overline{x}} \leq \min(n_{\overline{x}}, r_{\underline{z}}).$$

2 Likelihood computation in detail

SnappNet uses Algorithm 1 to compute the full likelihood of a network Ψ with respect to D_i, the data from marker i. The algorithm starts by initializing the data structures that will subsequently be used and then processes all nodes of the network Ψ using the rules presented in the main text. Rules 2, 3 and 4 are applied respectively in Algorithm 3, 4 and 5, together with suitable modifications of data structures.

The data structures are the following: ReadyNodesQ, a queue storing the nodes that are ready to be processed; Processed, which stores whether a node has already been processed or not; and CurrF, a dictionary that associates any branch x to the $\mathbf{F_x}$ having $\overline{x}$ in $\mathbf{x}$. In this pseudocode, $\mathbf{F_x}$ represents a data structure holding all the relevant values of $\mathbf{F_x}(\mathbf{n_x}, \mathbf{r_x})$, as well as the vector of population interfaces $\mathbf{x}$. We also note that, to reduce memory usage, we only store the $\mathbf{F_x}$ associated to branches that separate an unprocessed node to a processed node, as these are the only ones that will be used in future computations. Note that unlike in the main text, nodes are denoted u, u' and u_p in S1 Text.

Algorithm 1: Compute the likelihood for one marker

```
Input: Network Ψ, and the data D_i for one marker
Output: The likelihood ℙ(D_i|Ψ)

// Defining global data structures shared by all algorithms
Let ReadyNodesQ be an empty queue
Let CurrF and Processed be empty dictionaries
Initialize_Data_Structures(D_i)
while ReadyNodesQ ≠ ∅ do
    u ← Dequeue(ReadyNodesQ)
    if u has two outgoing branches e_1 and e_2 then // u is a tree node
        if CurrF[e_1] ≠ CurrF[e_2] then          // comparing pointers
            Apply_Rule_2(u)
        else Apply_Rule_4(u)
    else Apply_Rule_3(u)                       // u is a reticulation node
end
Let ρ be the root branch in Ψ
Compute ℙ(D_i|Ψ) from F_(ρ) using Equation (3)
return ℙ(D_i|Ψ)
```

Algorithm 2: `Initialize_Data_Structures`(D_i)

```
foreach leaf x̲ in Ψ do
    Compute n_x̲ and r_x̲ from D_i
    Compute F_(x̲) using Rule 0
    Compute F_(x̄) using Rule 1
    CurrF[x] ← F_(x̄)
    Processed[x̲] ← true
end
foreach internal node u in Ψ do
    Processed[u] ← false
    if all children of u are leaves then Enqueue (ReadyNodesQ,u)
end
```

Algorithm 3: `Apply_Rule_2`(u) // u is a tree node of Ψ

Let x, y be u's outgoing branches and let z be u's incoming branch

$\mathbf{F}_{\mathbf{x},\overline{x}} \leftarrow$ CurrF[x]

$\mathbf{F}_{\mathbf{y},\overline{y}} \leftarrow$ CurrF[y]

Apply Rule 2 to obtain $\mathbf{F}_{\mathbf{x},\mathbf{y},\underline{z}}$ from $\mathbf{F}_{\mathbf{x},\overline{x}}$ and $\mathbf{F}_{\mathbf{y},\overline{y}}$

if *u is the root node of Ψ* **then return**

Apply Rule 1 to obtain $\mathbf{F}_{\mathbf{x},\mathbf{y},\overline{z}}$ from $\mathbf{F}_{\mathbf{x},\mathbf{y},\underline{z}}$

foreach *branch w with an interface in* $\mathbf{x}, \mathbf{y}, \overline{z}$ **do**

  CurrF[w] $\leftarrow \mathbf{F}_{\mathbf{x},\mathbf{y},\overline{z}}$ // copying pointers only

Processed[u] $\leftarrow$ true

`CheckParentIsReady`(z)

Algorithm 4: `Apply_Rule_3`(u) // u is a reticulation node of Ψ

Let x be u's outgoing branch and let y, z be u's incoming branches
$\mathbf{F}_{\mathbf{x},\overline{x}} \leftarrow$ CURRF[x]
Apply Rule 3 to obtain $\mathbf{F}_{\mathbf{x},\underline{y},\underline{z}}$ from $\mathbf{F}_{\mathbf{x},\overline{x}}$
Apply Rule 1 twice to obtain $\mathbf{F}_{\mathbf{x},\overline{y},\overline{z}}$ from $\mathbf{F}_{\mathbf{x},\underline{y},\underline{z}}$
foreach *branch w with an interface in* $\mathbf{x}, \overline{y}, \overline{z}$ **do**
  CURRF[w] $\leftarrow \mathbf{F}_{\mathbf{x},\overline{y},\overline{z}}$ // copying pointers only
PROCESSED[u] $\leftarrow$ `true`
`CheckParentIsReady`(y)
`CheckParentIsReady`(z)

Algorithm 5: `Apply_Rule_4`(u) // u is a tree node of Ψ

Let x, y be u's outgoing branches and let z be u's incoming branch
// recall that here CURRF[x] = CURRF[y]
$\mathbf{F}_{\mathbf{z},\overline{x},\overline{y}} \leftarrow$ CURRF[x]
Apply Rule 4 to obtain $\mathbf{F}_{\mathbf{z},\underline{z}}$ from $\mathbf{F}_{\mathbf{z},\overline{x},\overline{y}}$
if u *is the root node of* Ψ **then return**
Apply Rule 1 to obtain $\mathbf{F}_{\mathbf{z},\overline{z}}$ from $\mathbf{F}_{\mathbf{z},\underline{z}}$
foreach *branch w with an interface in* $\mathbf{z}, \overline{z}$ **do**
  CURRF[w] $\leftarrow \mathbf{F}_{\mathbf{z},\overline{z}}$
PROCESSED[u] $\leftarrow$ `true`
`CheckParentIsReady`(z)

Algorithm 6: `CheckParentIsReady`(x_p) // x_p is a branch of Ψ

Result: Updated data structures, where the origin of x_p is added to READYNODESQ if all its descendants have already been processed

Let u_p and u be the nodes respectively at the origin and end of x_p
if u_p *has two parents* **then** // u_p is a reticulation node
  `Enqueue`(READYNODESQ,u_p)
else // u_p is a tree node
  Let u' be the child of u_p different from u
  if PROCESSED[u'] **then**
    `Enqueue` (READYNODESQ, u_p)
  end
end

3 Other computational complexity results

In this section, we shall use the weak definition of connectivity in a directed graph: we say that two nodes in Ψ are *connected* is there is an undirected path between them in Ψ. The same holds for the notion of *biconnected*, see below.

3.1 Time complexity of the algorithm by Zhu et al. [1]

Although the time complexity stated by Zhu and coauthors is $O(sn^{4r+4})$, where r is the number of reticulation nodes in the network, they also note that *all labelled partial likelihoods (LPLs) at a lowest articulation node can be merged into a single LPL, thus avoiding carrying forth all that information* [1]. This means that, as we stated in the main text, the time complexity to process a node with their algorithm is actually $O(n^{4r_u+4})$, where r_u is the number of reticulation nodes which descend from u, and for which there exists a directed path from u that does not pass via a lowest articulation node. Note that r_u is potentially much smaller than r. We refer to the original paper by Zhu and coauthors for the definition of LPL and the full description of their algorithm [1].

Here we prove that, since the time complexity to process a node is $O(n^{4r_u+4})$, then the whole algorithm runs in $O(sn^{4\ell+4})$ time, where ℓ is the *level* of the network [5, 6].

Let us first recall some definitions from the theory of phylogenetic networks that are fundamental to analyse the complexity of the algorithm by Zhu et al. [1]. A subgraph G of Ψ is *biconnected* if the removal of any one node in G leaves the remainder of G connected. A *biconnected component* of Ψ is a maximal biconnected subgraph of Ψ. The nodes of Ψ that belong to two or more biconnected components are called *articulation nodes*. (Equivalently, articulation nodes are the nodes in Ψ whose removal cause the network to become disconnected.) An articulation node is said to be a *lowest articulation node* if all of its children are not articulation nodes. The *level* of a phylogenetic network is the maximum number of reticulation nodes in one of its biconnected components.

It is easy to see that a phylogenetic network has two kinds of biconnected components: those that only consist of two adjacent nodes — which we call *trivial* biconnected components — and more complex ones — which we call nontrivial biconnected components or *blobs*. Every articulation node of Ψ is found at the root of a biconnected component. The lowest articulation nodes of a network coincide with the roots of the network's blobs.

Recall that r_u is defined as the number of reticulation nodes which descend from u, and for which there exists a directed path from u that does not pass via a lowest articulation node. Now note that every directed path that ends in a reticulation node v and does not pass via a lowest articulation node can only be from a node u in the same blob as v. Then, r_u is at most equal to the

number of reticulation nodes in the same biconnected component as u. In turn, the number of reticulation nodes in the same biconnected component as u is at most equal to ℓ, the level of Ψ. We can then conclude that $r_u \leq \ell$ and that each node is processed in at most $O(n^{4\ell+4})$ time, giving a total running time of $O(sn^{4\ell+4})$.

3.2 SnappNet's $\overline{K}$ and the level of the network

Here we prove that for any traversal of the network Ψ, we have $\overline{K} \leq \ell+1$, where ℓ is the level of Ψ (Proposition 1 below).

We let $B(\mathbf{x})$ denote the set of branches x for which there exists a population interface $\underline{x}$ or $\overline{x}$ in the VPI $\mathbf{x}$. Moreover we let $G^{\Psi}_{\mathbf{x}}$ denote the subgraph of Ψ induced by all the descendant nodes of the branches in $B(\mathbf{x})$.

The intuition behind the proof is that, for any VPI activated by the traversal algorithm, the branches in $B(\mathbf{x})$ must all belong to the same biconnected component of Ψ. Moreover, $|B(\mathbf{x})|$ cannot exceed 1 + the number of reticulations within that biconnected component, which implies $\overline{K} \leq \ell + 1$.

Lemma 1. *Let $\mathbf{x}$ be a VPI activated by any traversal algorithm using Rules 0-4. Then, $G^{\Psi}_{\mathbf{x}}$ is connected.*

Proof. If $\mathbf{x} = (\underline{x})$ is activated by Rule 0, then $G^{\Psi}_{\mathbf{x}}$ consists of a single leaf and is trivially connected. Thus, we just need to prove that every subsequent application of Rules 1-4 can only activate a VPI $\mathbf{x}$ with connected $G^{\Psi}_{\mathbf{x}}$, assuming that this property is satisfied by the VPI or VPIs that the rule uses as input.

For Rule 1, this is trivially true as $G^{\Psi}_{\mathbf{x},\overline{x}} = G^{\Psi}_{\mathbf{x},\underline{x}}$. For Rule 2, let's assume that $G^{\Psi}_{\mathbf{x},\overline{x}}$ is connected and that $G^{\Psi}_{\mathbf{y},\overline{y}}$ is connected. This implies that $G^{\Psi}_{\mathbf{x},\mathbf{y},\underline{z}}$ is connected, as x and y appear in $G^{\Psi}_{\mathbf{x},\mathbf{y},\underline{z}}$ and ensure that all nodes in $G^{\Psi}_{\mathbf{x},\overline{x}}$ are connected to all nodes in $G^{\Psi}_{\mathbf{y},\overline{y}}$. For Rule 3 and 4, the thesis is again trivial, because $G^{\Psi}_{\mathbf{x}}$ for the newly active VPI only differs from the one for the input VPI by inclusion of a single new vertex, which is easily seen to be connected to the rest of $G^{\Psi}_{\mathbf{x}}$. □

Corollary 1. *Let $\mathbf{x}$ be a VPI activated by any traversal algorithm using Rules 0-4. Then, all the branches in $B(\mathbf{x})$ belong to the same biconnected component of Ψ.*

Proof. If $|B(\mathbf{x})| = 1$, this is trivial. If $B(\mathbf{x})$ contains at least two branches x and y, it is now easy to see that x and y belong to a cycle obtained by attaching the following two disjoint paths: (1) the path within $G^{\Psi}_{\mathbf{x}}$ from the bottom of x to the bottom of y — which exists because of Lemma 1 — and (2) the path from the bottom of x to the bottom of y, going via x and y and only using branches that are ancestral to x and y. The existence of this cycle implies the thesis. □

Lemma 2. *Let* $\mathbf{x}$ *be a VPI activated by any traversal algorithm using Rules 0-4, and let* $R(\mathbf{x})$ *be the set of reticulation nodes that descend from any branch in* $B(\mathbf{x})$ *and belong to the same biconnected component as the one of* $B(\mathbf{x})$*. Then,* $|B(\mathbf{x})| \leq |R(\mathbf{x})| + 1$*.*

Proof. To make notation light, let $b(\mathbf{x}) = |B(\mathbf{x})|$ and $r(\mathbf{x}) = |R(\mathbf{x})|$. As in the proof of Lemma 1, we start by noting that if $\mathbf{x} = (\underline{x})$ is activated by Rule 0, then the thesis trivially holds, as $b((\underline{x})) = 1$ and $r((\underline{x})) = 0$.

We then consider the other rules, and show that if the thesis holds for the VPIs that have already been activated, then it must hold for the newly activated VPI. For Rule 1, $b(\mathbf{x}, \overline{x}) = b(\mathbf{x}, \underline{x})$ and $r(\mathbf{x}, \overline{x}) = r(\mathbf{x}, \underline{x})$, so $b(\mathbf{x}, \underline{x}) \leq r(\mathbf{x}, \underline{x}) + 1$ trivially implies $b(\mathbf{x}, \overline{x}) \leq r(\mathbf{x}, \overline{x}) + 1$.

For Rule 2, we assume $b(\mathbf{x}, \overline{x}) \leq r(\mathbf{x}, \overline{x}) + 1$ and $b(\mathbf{y}, \overline{y}) \leq r(\mathbf{y}, \overline{y}) + 1$. Now note that $b(\mathbf{x}, \mathbf{y}, \underline{z}) = b(\mathbf{x}, \overline{x}) + b(\mathbf{y}, \overline{y}) - 1$, and $r(\mathbf{x}, \mathbf{y}, \underline{z}) = r(\mathbf{x}, \overline{x}) + r(\mathbf{y}, \overline{y})$ which imply:

$$\begin{aligned} b(\mathbf{x}, \mathbf{y}, \underline{z}) &= b(\mathbf{x}, \overline{x}) + b(\mathbf{y}, \overline{y}) - 1 \\ &\leq (r(\mathbf{x}, \overline{x}) + 1) + (r(\mathbf{y}, \overline{y}) + 1) - 1 \\ &= r(\mathbf{x}, \overline{x}) + r(\mathbf{y}, \overline{y}) + 1 \\ &= r(\mathbf{x}, \mathbf{y}, \underline{z}) + 1, \end{aligned}$$

thus proving the thesis for VPI $\mathbf{x}, \mathbf{y}, \underline{z}$.

For Rule 3, we assume $b(\mathbf{x}, \overline{x}) \leq r(\mathbf{x}, \overline{x}) + 1$. Now note that

$$\begin{aligned} b(\mathbf{x}, \underline{y}, \underline{z}) &= b(\mathbf{x}, \overline{x}) + 1, \\ r(\mathbf{x}, \underline{y}, \underline{z}) &= r(\mathbf{x}, \overline{x}) + 1, \end{aligned}$$

which implies $b(\mathbf{x}, \underline{y}, \underline{z}) \leq r(\mathbf{x}, \underline{y}, \underline{z}) + 1$.

Finally, for Rule 4, we assume $b(\mathbf{z}, \overline{x}, \overline{y}) \leq r(\mathbf{z}, \overline{x}, \overline{y}) + 1$. Now distinguish between two cases. Either (i) $\mathbf{z}$ is nonempty, in which case $B(\mathbf{z}, \overline{x}, \overline{y})$ and $B(\mathbf{z}, \underline{z})$ are in the same biconnected component and

$$\begin{aligned} b(\mathbf{z}, \underline{z}) &= b(\mathbf{z}, \overline{x}, \overline{y}) - 1, \\ r(\mathbf{z}, \underline{z}) &= r(\mathbf{z}, \overline{x}, \overline{y}). \end{aligned}$$

In this case we therefore have $b(\mathbf{z}, \underline{z}) \leq r(\mathbf{z}, \underline{z})$, which implies the thesis.

Alternatively, (ii) $\mathbf{z}$ is empty, in which case

$$\begin{aligned} b(\mathbf{z}, \underline{z}) &= 1, \\ r(\mathbf{z}, \underline{z}) &= 0. \end{aligned}$$

Thus $b(\mathbf{z}, \underline{z}) \leq r(\mathbf{z}, \underline{z}) + 1$ is again satisfied. □

We now have all we need to prove the main result of this section:

Proposition 1. *For any traversal algorithm using Rules 0-4 to process a network of level ℓ, $\overline{K} \leq \ell + 1$.*

Proof. Note that

$$\overline{K} = \max\{|B(\mathbf{x})| \text{ such that } \mathbf{x} \text{ is activated by the given traversal algorithm}\}.$$

Thus, using Lemma 2, and the definition of the level ℓ:

$$\begin{aligned}\overline{K} &\leq \max\{|R(\mathbf{x})| + 1 \text{ such that } \mathbf{x} \text{ is activated by the given traversal algorithm}\} \\ &\leq \ell + 1.\end{aligned}$$

□

4 Newick representations

Network A:
((C:0.08,((R:0.007,(Q:0.004)#H1:0.003):0.035,((A:0.006,#H1:0.002):0.016,
L:0.022):0.02):0.038):0);

Network B:
((((R:0.014,(Q:0.004)#H1:0.01):0.028,(((A:0.003)#H2:0.003,#H1:0.002)
:0.016,L:0.022):0.02):0.038,(C:0.005,#H2:0.002):0.075):0);

Network C:
((O:0.08,((A:0.012,((B:0.002,(C:0.001)#H1:0.001):0.002)#H2:0.008):0.038,
((D:0.003,#H1:0.002):0.017,#H2:0.016):0.03):0.03):0);

Starting tree for networks A and B:
(((C:0.05,R:0.05):0.05,((A:0.05,L:0.05):0.025,Q:0.075):0.025):0);

Alternative starting trees for networks A and B (only used to check the influence of the starting tree):
(((A:0.05,Q:0.05):0.05,((C:0.05,L:0.05):0.025,R:0.075):0.025):0);
((((C:0.05,A:0.05):0.05,((R:0.05,Q:0.05):0.025,L:0.075):0.025):0);

Starting tree for network C:
(((O:0.05,A:0.05):0.05,((C:0.05,D:0.05):0.025,B:0.075):0.025):0);

5 `MCMCBiMarkers` commands

For m=100,000 , data were generated in the following way:

```
SimBiMarkersinNetwork -pi0 0.5 -sd 17000 -num 100000
-tm <A:A_0;B:B_0,B_1,B_2,B_3;
C:C_0,C_1,C_2,C_3;D:D_0;O:O_0>
-truenet "[0.005](O:0.08:0.005,((A:0.012:0.005,((B:0.002:0.005,
(C:0.001:0.005)I1#H1:0.001:0.005:0.5)I2:0.002:0.005)I3#H2:0.008
:0.005:0.5)I4:0.038:0.005,((D:0.003:0.005,
I1#H1:0.002:0.005:0.5)I5:0.017:0.005,I3#H2:0.016:0.005:0.5)
I6:0.03:0.005)I7:0.03:0.005);"
;
```

Next, the following commands, were successively used to run `MCMCBiMarkers`. The first step consists in a pre-burnin phase relying on 3 chains of different temperatures.

```
MCMC_BiMarkers -cl 1500000 -sf 1000 -bl 200000 -prebl 10000
-premc3 (2.0,4.0) -premr 1 -pi0 0.5 -varytheta
-pp 2.0 -ee 2.0 -mr 2
```

```
-pl 1
-esptheta -sd 12345678
-taxa (A_0,B_0,B_1,B_2,B_3,C_0,C_1,C_2,C_3,D_0,O_0)
-tm <A:A_0;B:B_0,B_1,B_2,B_3;C:C_0,C_1,C_2,C_3;D:D_0;O:O_0>
;
```

The second step consists in MCMC sampling during 1.5×10^6 iterations.

```
MCMC_BiMarkers -cl 1500000 -sf 1000 -bl 200000
-pi0 0.5 -varytheta
-pp 2.0 -ee 2.0 -mr 2
-pl 1
-esptheta -sd 12345678
-taxa (A_0,B_0,B_1,B_2,B_3,C_0,C_1,C_2,C_3,D_0,O_0)
-tm <A:A_0;B:B_0,B_1,B_2,B_3;C:C_0,C_1,C_2,C_3;D:D_0;O:O_0>
-snet"..."
;
```

Note that the "-snet" option refers to the starting network obtained from the pre-burnin phase. Besides, the options "-mr" and "-pp" allow to specify the network prior: the maximum number of reticulations was set to 2, and the prior Poisson distribution on the number of reticulation nodes was centered on 2.

6 Supplementary results for the simulation study

Table A. Table linked to Table 1 of the main manuscript. Trees inferred by SnappNet when m=1,000 sites were considered.

**Hyperparameters**	**Network A**	**Network B**
**True** ($\alpha = 1$, $\beta = 200$, $\frac{\alpha}{\beta} = 0.005$)	78.71% tree ((((Q,A),L),R),C)	35.28% tree (((Q,R),L),(A,C))<br>28.54% tree (((Q,L),R),(A,C))
**True** ($\alpha = 1$, $\beta = 1000$, $\frac{\alpha}{\beta} = 0.001$)	82.82% tree ((((Q,A),L),R),C)	45.27% tree (((Q,R),L),(A,C))<br>40.35% tree (((Q,L),R),(A,C))
**True** ($\alpha = 1$, $\beta = 2000$, $\frac{\alpha}{\beta} = 5 \times 10^{-4}$)	82.92% tree ((((Q,A),L),R),C)	48.40% tree (((Q,R),L),(A,C))<br>38.16% tree (((Q,L),R),(A,C))

Table B. Average posterior probability (PP) of the topology of network C obtained by running `MCMCBiMarkers` on data simulated from network C. Same as Table 3 of the main manuscript except that 12×10^6 iterations are considered, and only one lineage is sampled in hybrid species B and C. $\overline{\text{ESS}}$ is the average ESS over the different replicates, and SE stands for the sampler efficiency.

	**Number of sites**		
	**1,000**	**10,000**	**100,000**
**PP**	5.5×10^{-6} (20 replicates)	5.10% (19 replicates)	0% (16 replicates)
**SE**	2.32×10^{-5}	8.11×10^{-6}	1.96×10^{-5}
$\overline{\textbf{ESS}}$	250.88	87.63	211.57

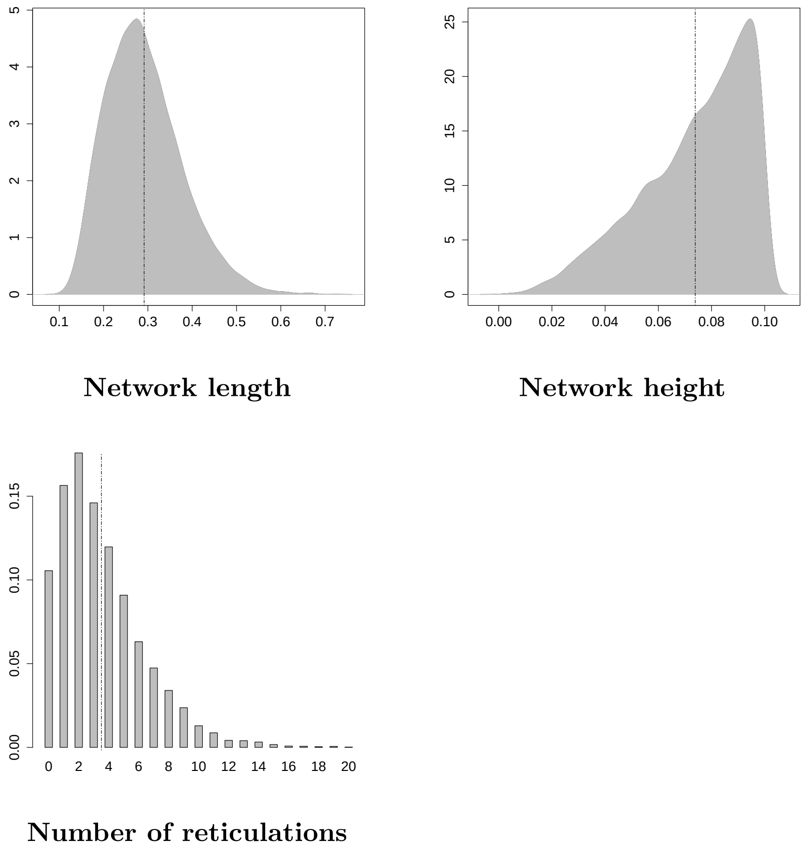

Figure A. Density probabilities for 5-tips networks, simulated with a prior corresponding to a birth hybridization process with parameters $d = 10$, $r = 1/2$ and $\tau_0 = 0.1$, using the SpeciesNetwork package [7]. The figure is obtained for 10,000 replicates. The means are given by the dashed vertical lines.

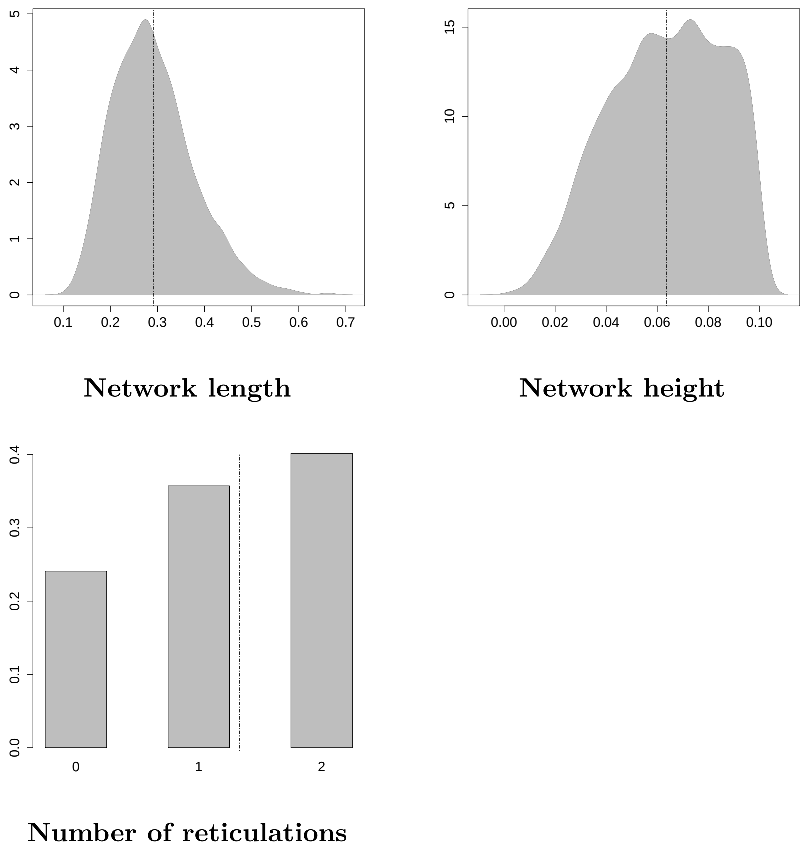

Figure B. Density probabilities for 5-tips networks with at most two reticulations, simulated with a prior corresponding to a birth hybridization process with parameters $d = 10$, $r = 1/2$ and $\tau_0 = 0.1$, using the SpeciesNetwork package [7]. Figures are drawn for the 4,377 cases in 10,000 where the network had at most two reticulations. The means are given by the dashed vertical lines.

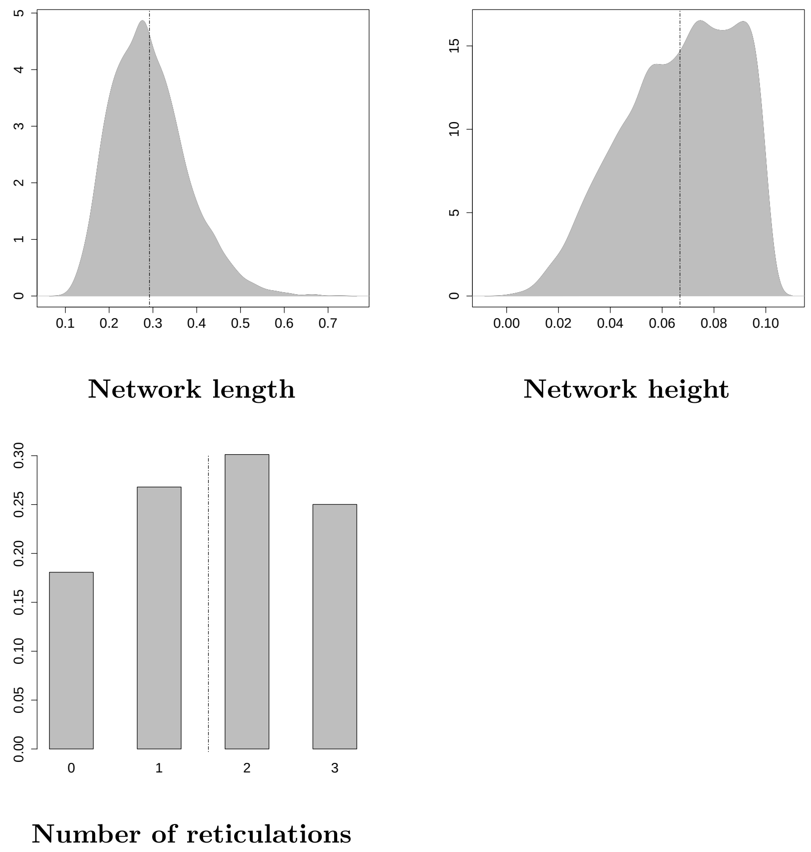

Figure C. Density probabilities regarding the 5-tips network with a maximum of 3 reticulations, simulated under the birth hybridization process ($d = 10$, $r = 1/2$, $\tau_0 = 0.1$, 5,837 replicates), using the SpeciesNetwork package [7]. The means are given by the dashed vertical lines.

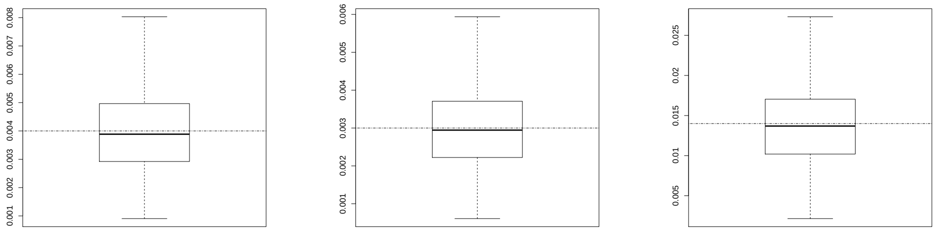

Height of H_1 **Height of H_2** **Height of MRCA(Q,R)**

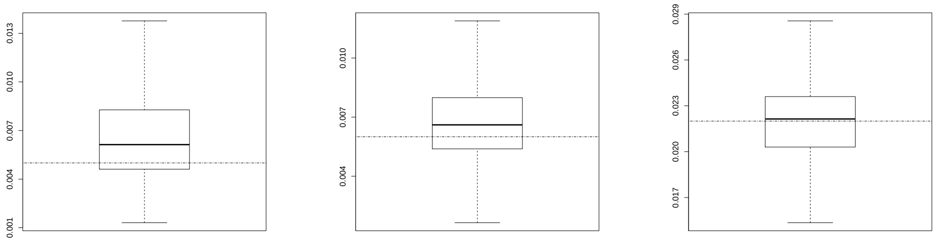

Height of MRCA(C,A) **Height of MRCA(H_1,H_2)** **Height of MRCA(Q,L)**

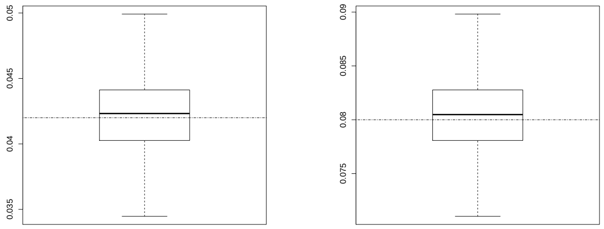

Height of MRCA(L,R) **Height of MRCA(C,R)**

Figure D. Estimated node heights of network B. 10,000 sites are considered and 2 lineages per species. Constant sites are included in the analysis, and the estimated heights are based on the 12 replicates (over 14 replicates) for which network B was recovered by SnappNet (criterion ESS> 200 ; $\theta \sim \Gamma(1, 200)$, $d \sim \mathcal{E}(0.1)$, $r \sim \text{Beta}(1, 1)$, $\tau_0 \sim \mathcal{E}(10)$ for the priors, number of reticulations bounded by 3 when exploring the network space). Heights are measured in units of expected number of mutations per site. True values are given by the dashed horizontal lines. The initials MRCA stand for "Most Recent Common Ancestor".

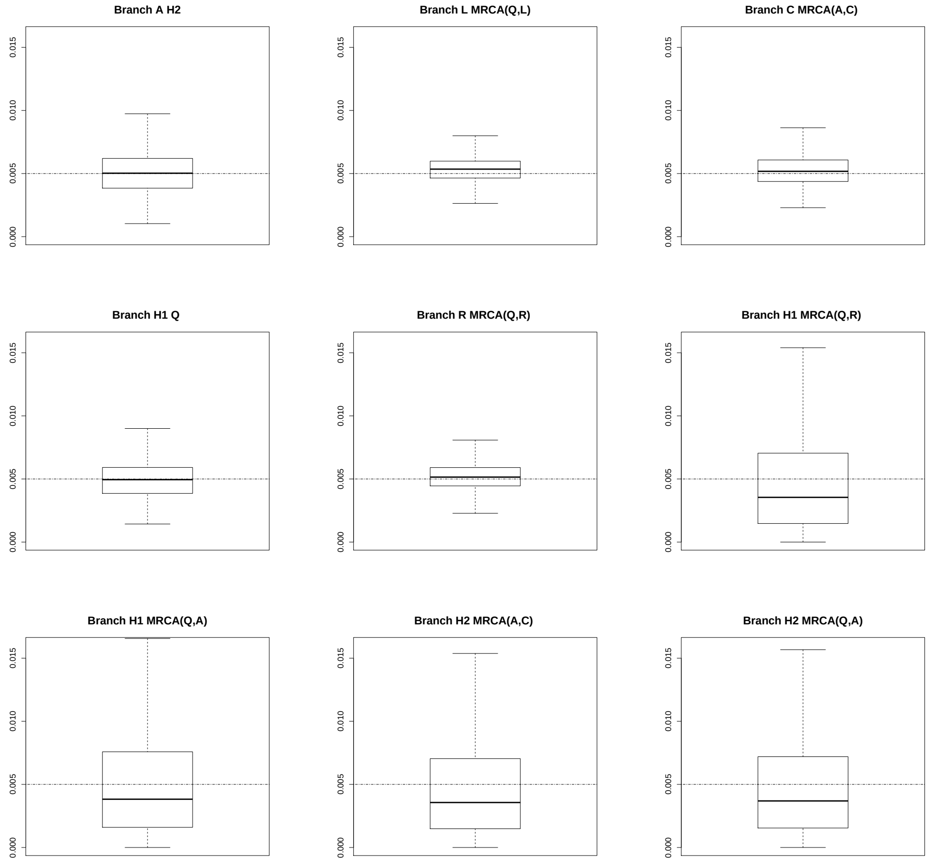

Figure E. Estimated population sizes θ for each branch of network B. 10,000 sites are considered and 2 lineages per species. Same framework as Figure D. True values are given by the dashed horizontal lines. The initials MRCA stand for "Most Recent Common Ancestor".

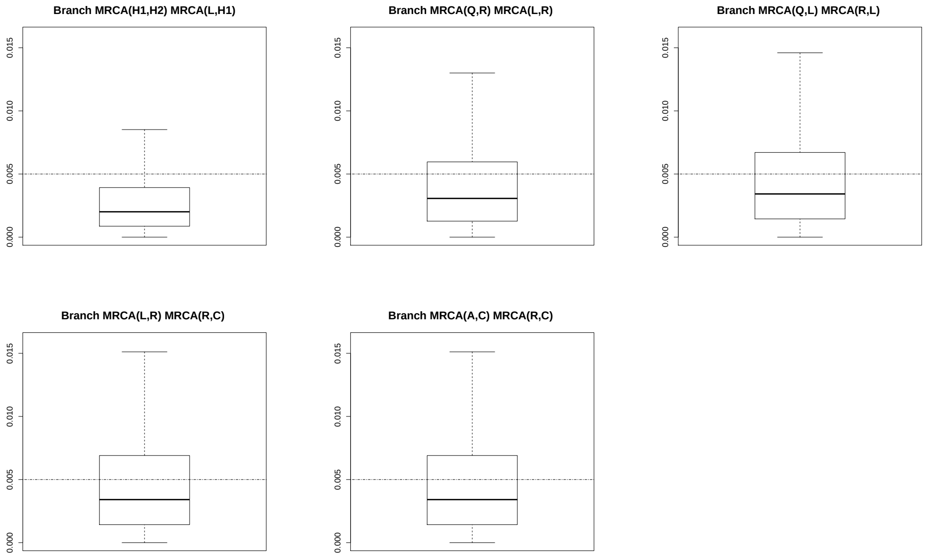

Figure F. Same framework as Figure E.

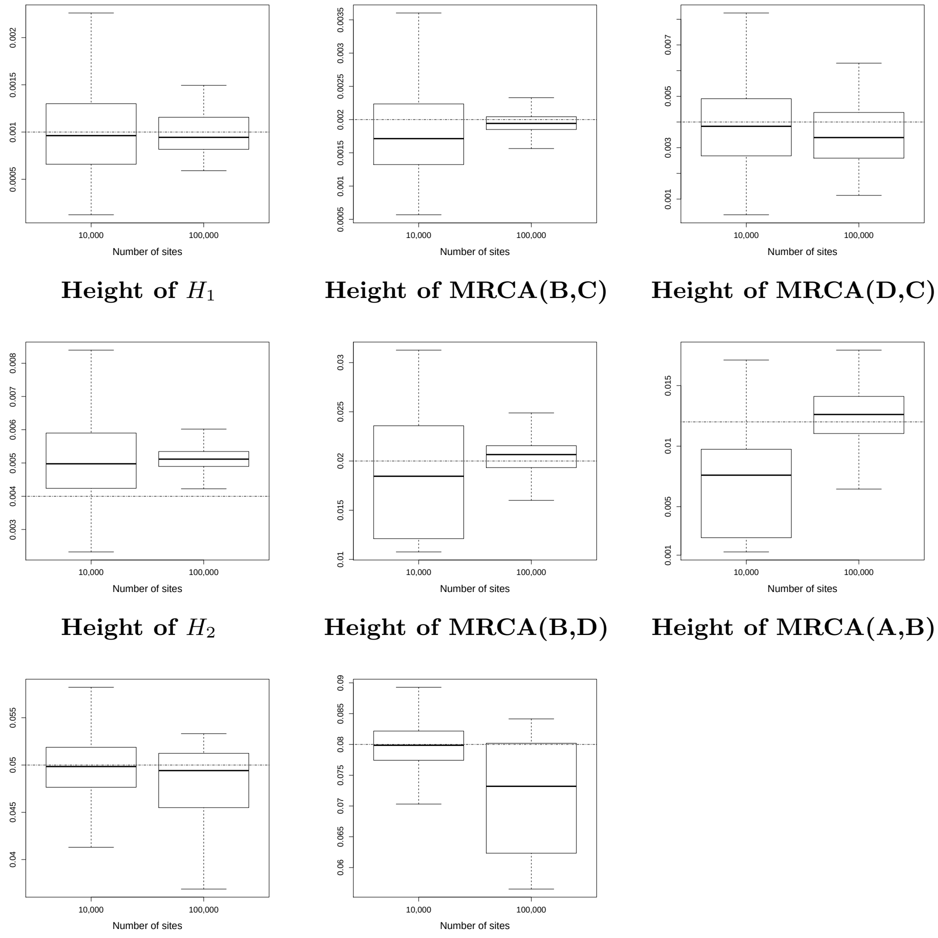

Height of H_1 **Height of MRCA(B,C)** **Height of MRCA(D,C)**

Height of H_2 **Height of MRCA(B,D)** **Height of MRCA(A,B)**

Height of MRCA(A,D) **Height of MRCA(O,D)**

Figure G. Estimated node heights of network C as a function of the number of sites. Same experiment as in Table 2 of the main manuscript: 1 lineage in species O, A and D, and 4 lineages in species B and C. The estimated heights are based on the replicates for which network C was recovered by SnappNet. True values are given by the dashed horizontal lines. The initials MRCA stand for "Most Recent Common Ancestor".

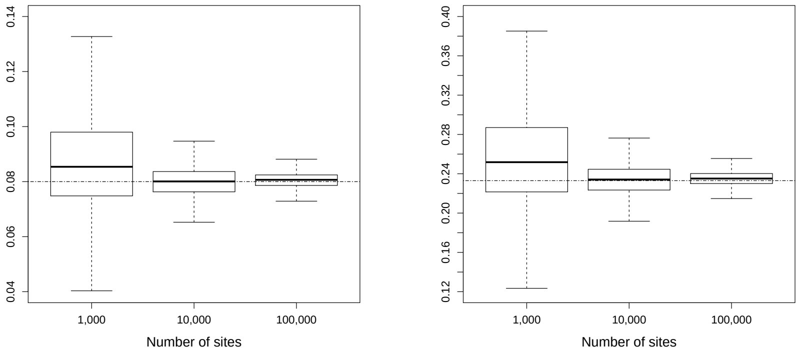

Network height **Network length**

Figure H. Estimated height and length for network A, as a function of the number of sites. Heights and lengths are measured in units of expected number of mutations per site. True values are given by the dashed horizontal lines. Two lineages per species were simulated. Only polymorphic sites are included in the analysis, and 20 replicates are considered for each simulation set up (criterion ESS> 200 for m=1,000 and m=10,000 , and criterion ESS> 100 for m=100,000; $\theta \sim \Gamma(1, 200)$, $d \sim \mathcal{E}(0.1)$, $r \sim \mathrm{Beta}(1, 1)$, $\tau_0 \sim \mathcal{E}(10)$ for the priors, number of reticulations bounded by 2 when exploring the network space). Same framework as in Figure 10 of the main paper, except that only polymorphic sites are taken into account.

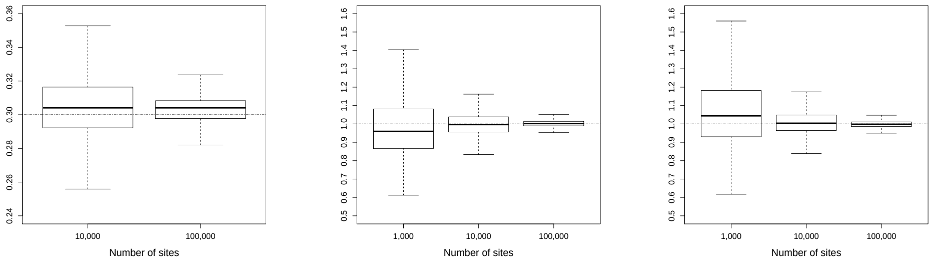

Inheritance probability γ **Instantaneous rate** u **Instantaneous rate** v

Figure I. Estimated inheritance probability and instantaneous rates for network A, as a function of the number of sites. True values are given by the dashed horizontal lines. Same framework as in Figure 11 of the main paper, except that only polymorphic sites are taken into account.

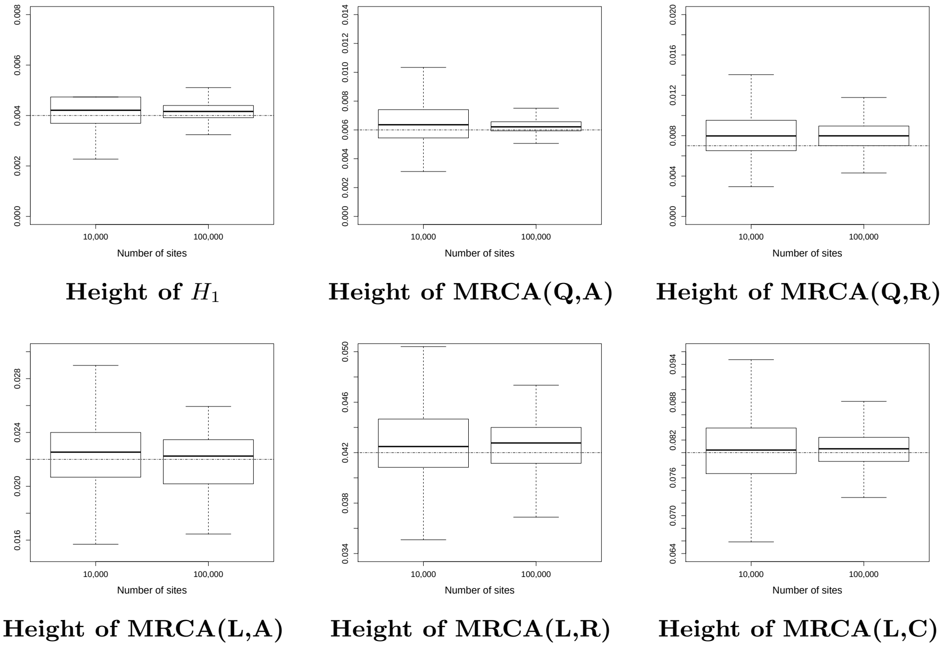

Figure J. Estimated node heights of network A, as a function of the number of sites. Heights are measured in units of expected number of mutations per site. True values are given by the dashed horizontal lines. Same framework as in Figure 12 of the main paper, except that only polymorphic sites are taken into account. The initials MRCA stand for "Most Recent Common Ancestor".

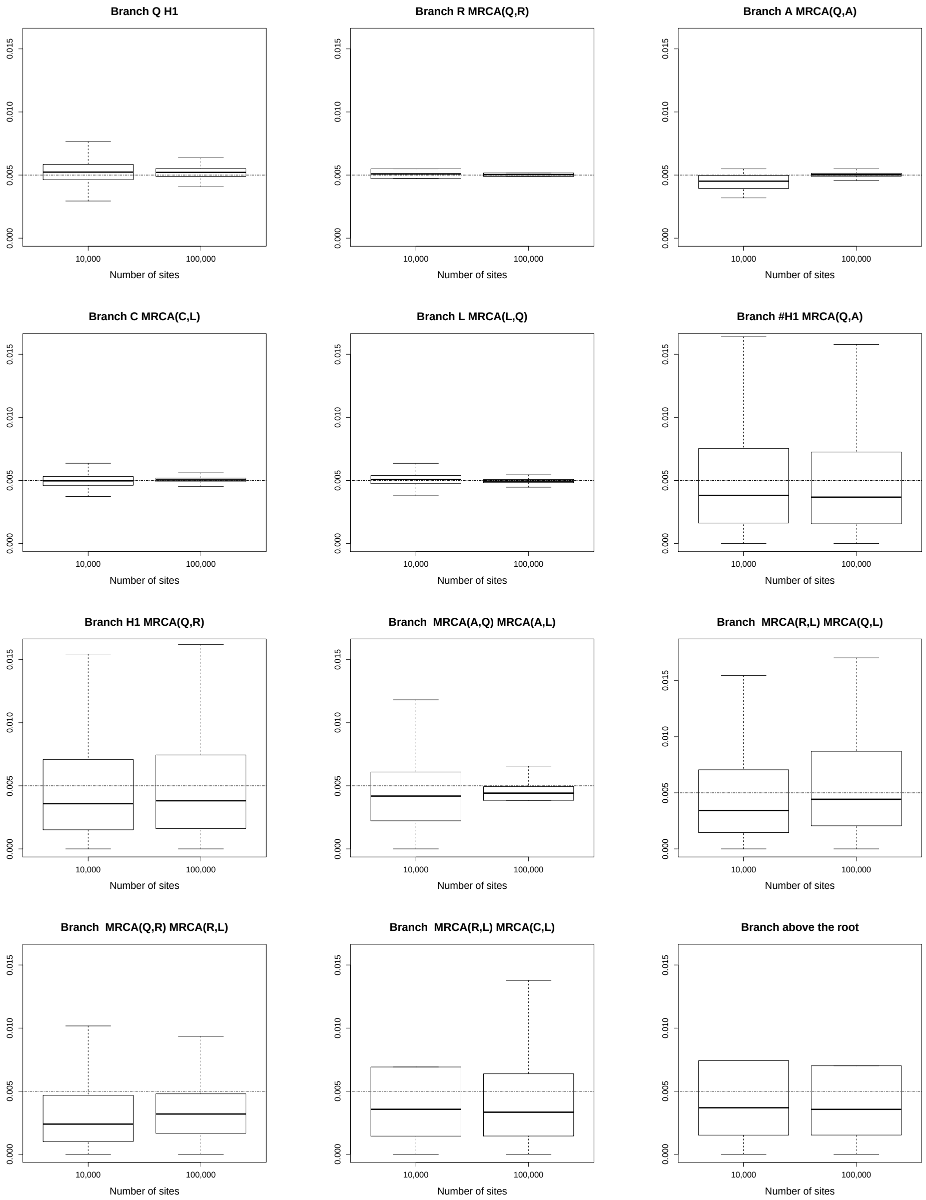

Figure K. Estimated population sizes θ for each branch of network A, as a function of the number of sites. True values are given by the dashed horizontal lines. Same framework as in Figure 13 of the main paper, except that only polymorphic sites are taken into account. The initials MRCA stand for "Most Recent Common Ancestor".

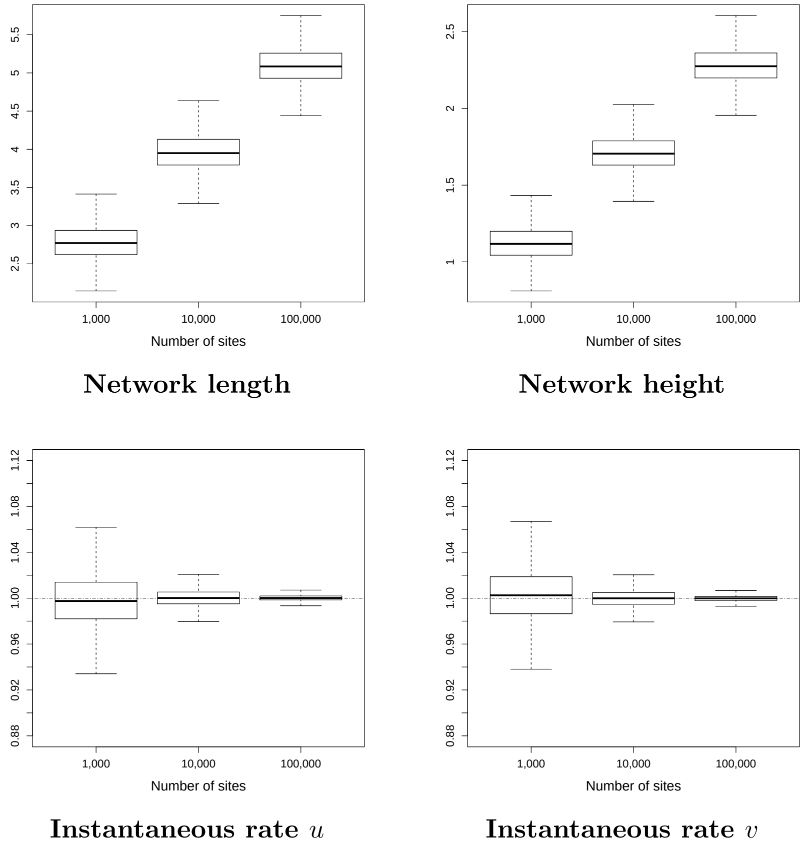

Figure L. Experiments on Network A and based only on polymorphic sites. Same framework as in Figures H and I above, except that the correction factor is not used in the calculations (criterion ESS>200 in all cases).

7 Supplementary informations on rice real data

Table C. Description of the 24 rice varieties considered in our study. These varieties are either representative cultivars spanning the four main rice subpopulations (Indica, Japonica, *circum* Aus and *circum* Basmati), or wild types (Or1I, Or1A, Or3).

Subpopulation	Variety ID	Country	Variety name
circum Aus	IRIS-313-11058	Bangladesh	AUS 329
	IRIS 313-11737	India	CHUNDI
	IRIS-313-10852	India	ARC 7336
	IRIS-313-11027	Pakistan	JHONA 101
circum Basmati	IRIS-313-11062	Bangladesh	BEGUNBICHI 33
	IRIS-313-8326	India	JC1
	IRIS-313-11258	India	ARC 13502
	IRIS-313-12094	Bangladesh	ARC KASHA
Indica	IRIS-313-11819	Myanmar	PADINTHUMA
	IRIS-313-11089	Cambodia	SRAU THMOR
	IS-313-11646	India	NCS771 A
	IRIS-313-11741	SriLanka	HERATH BANDA
Japonica	B204	China	LONGHUAMAOHU
	IRIS-313-10577	Philippines	IFUGAO RICE
	IRIS-313-11691	Bhutan	SHANGYIPA
	IRIS-313-7883	Indonesia	GANIGI
	B269	China	YUEFU
Or1I	W1117	India	W1117
	W1559	Thailand	W1559
Or1A	W0574	Malaya	W0574
	W1747	India	W1747
Or3	W3042	China	W3042
	W3073	China	W3073
	W3048	China	W3048

Table D. Data set 1, that includes only one variety per subpopulation. These varieties were chosen from Table C.

Subpopulation	Variety ID	Country	Variety name
circum Aus	IRIS-313-10852	India	ARC 7336
circum Basmati	IRIS-313-12094	Bangladesh	ARC KASHA
Indica	IRIS-313-11741	SriLanka	HERATH BANDA
Japonica	B269	China	YUEFU
Or1I	W1559	Thailand	W1559
Or1A	W0574	Malaya	W0574
Or3	W3073	China	W3073

Table E. Data sets 2 and 3, that include two varieties per subpopulation. These varieties were chosen from Table C.

Subpopulation	Data set	Variety ID	Country	Variety name
circum Aus	2	IRIS-313-11058<br>IRIS-313-10852	Bangladesh<br>India	AUS 329<br>ARC 7336
	3	IRIS 313-11737<br>IRIS-313-11027	India<br>Pakistan	CHUNDI<br>JHONA 101
circum Basmati	2	IRIS-313-11062<br>IRIS-313-11258	Bangladesh<br>India	BEGUNBICHI 33<br>ARC 13502
	3	IRIS-313-8326<br>IRIS-313-12094	India<br>Bangladesh	JC1<br>ARC KASHA
Indica	2	IRIS-313-11819<br>IS-313-11646	Myanmar<br>India	PADINTHUMA<br>NCS771 A
	3	IRIS-313-11741<br>IRIS-313-11089	SriLanka<br>Cambodia	HERATH BANDA<br>SRAU THMOR
Japonica	2	B204<br>IRIS-313-11691	China<br>Bhutan	LONGHUAMAOHU<br>SHANGYIPA
	3	IRIS-313-10577<br>IRIS-313-7883	Philippines<br>Indonesia	IFUGAO RICE<br>GANIGI
Or1I	2,3	W1117<br>W1559	India<br>Thailand	W1117<br>W1559
Or1A	2,3	W0574<br>W1747	Malaya<br>India	W0574<br>W1747
Or3	2	W3042<br>W3073	China<br>China	W3042<br>W3073
	3	W3048<br>W3073	China<br>China	W3048<br>W3073

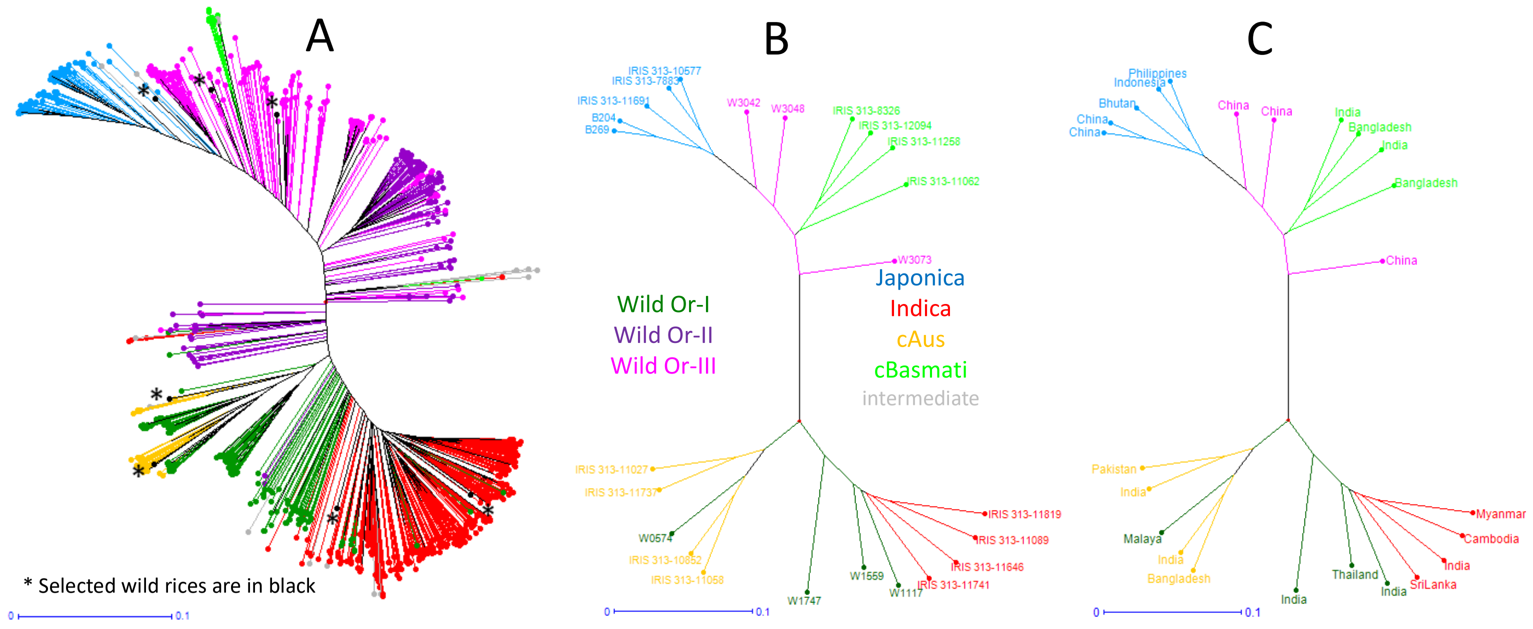

Figure M. Summary of rice molecular diversity used for selecting our sample of rice cultivated varieties and wild types. A: unweighted neighbour joining (UWNJ) tree reflecting dissimilarities among 899 accessions based on 2.48 million SNPs as described in [8]; the accessions are colored according to their classification into wild population types or cultivar groups. B and C: UWNJ tree using the same data for the 24 accessions we selected for assessing SnappNet performance, and showing their accessions number (B) and their country of origin (C); the colors are as in A.

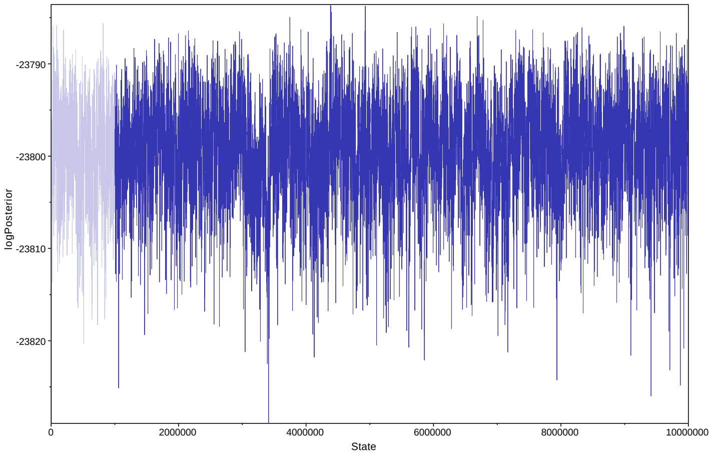

(a) First sampling, First chain

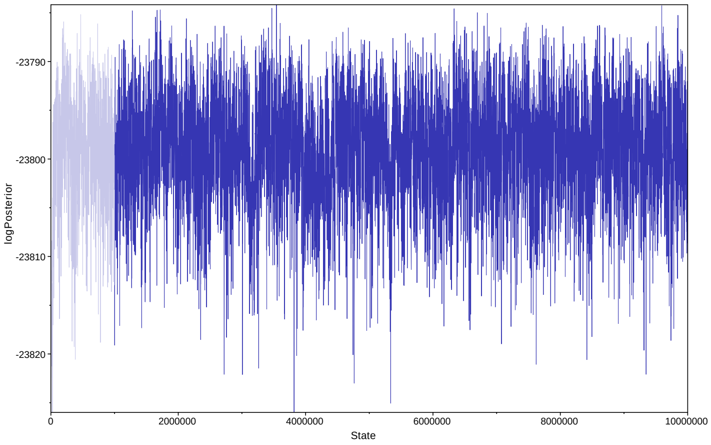

(b) First sampling, Second chain

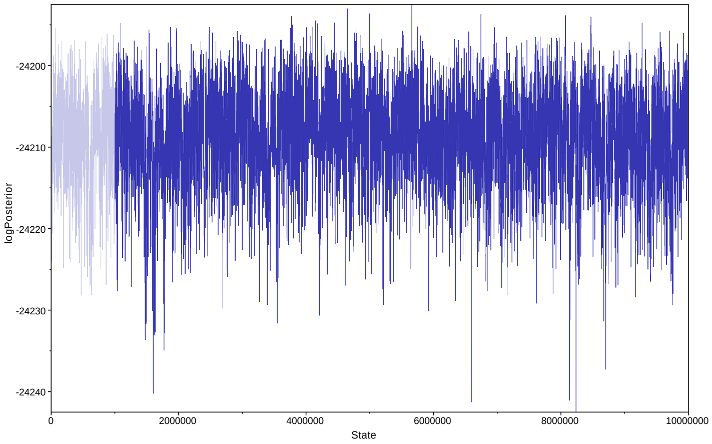

(c) Second sampling, First chain

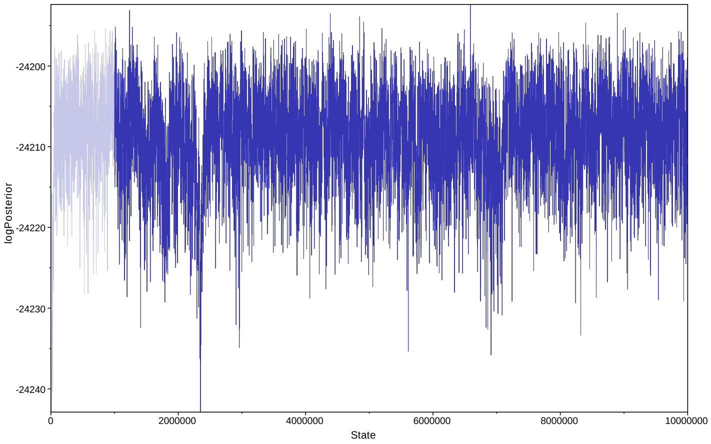

(d) Second sampling, Second chain

Figure N. Trace plots obtained according to the Tracer software when data set 1 was analyzed with SnappNet. (a) and (b) refer to the first sampling of 12 kSNPs along the whole genome, whereas (c) and (d) focus on the second sampling. Two chains were considered for each sampling.

Table F. Informations obtained according to the Tracer software, when data set 1 was analyzed with SnappNet. Two different samplings of 12 kSNPs were considered, and also two chains for each sampling.

		First Sampling		Second Sampling	
		**Chain 1**	**Chain 2**	**Chain 1**	**Chain 2**
**LogPosterior**	mean	-23799.1709	-23798.9118	-24208.6649	-24208.8018
	stdev	5.7064	5.5892	5.867	5.9986
	median	-23798.6288	-23798.4253	-24208.1221	-24208.2887
	auto-correlation time	10667.9058	7764.256	9263.5725	16818.357
	effective sample size	843.7	1159.3	971.7	535.2
**LogLikelihood**	mean	-23610.9374	-23610.7083	-24021.9798	-24021.9297
	stdev	4.1712	4.0226	3.9336	4.0567
	median	-23610.6061	-23610.3848	-24021.6408	-24021.5582
	auto-correlation time	34252.7407	28373.4116	31661.3188	68782.4427
	effective sample size	262.8	317.2	284.3	130.9
**LogPrior**	mean	-188.2335	-188.2035	-186.6851	-186.8721
	stdev	5.7941	5.4802	5.3762	5.4702
	median	-187.8239	-187.7722	-186.2485	-186.3268
	auto-correlation time	17357.6687	11954.8279	9509.4182	15383.8939
	effective sample size	518.6	752.9	946.5	585.1
$\boldsymbol{u}$	mean	0.5567	0.5567	0.5583	0.5583
	stdev	9.4491E-4	9.5177E-4	9.7888E-4	9.5855E-4
	median	0.5567	0.5567	0.5583	0.5582
	auto-correlation time	1898.9027	2043.8061	1913.3736	1932.747
	effective sample size	4740.1	4404	4704.3	4657.1
$\boldsymbol{v}$	mean	4.9094	4.9073	4.7922	4.7909
	stdev	0.0734	0.0739	0.0721	0.0706
	median	4.9072	4.9062	4.7903	4.7925
	auto-correlation time	1896.7939	2044.0868	1944.4961	1936.7774
	effective sample size	4745.4	4403.4	4629	4647.4
$\boldsymbol{d}$	mean	10.7192	10.9194	9.8551	10.0755
	stdev	5.2274	5.3009	4.7636	4.8326
	median	10.0307	10.2443	9.2427	9.4077
	auto-correlation time	3692.7762	2098.2587	5591.047	4875.9969
	effective sample size	2437.5	4289.7	1609.9	1846
$\boldsymbol{r}$	mean	0.2387	0.2349	0.2217	0.2159
	stdev	0.1707	0.1667	0.1633	0.1558
	median	0.1996	0.1952	0.1799	0.179
	auto-correlation time	6276.9007	2088.925	1786.1765	1610.1668
	effective sample size	1434	4308.9	5039.3	5590.1

8 Additional experiments on SnappNet's MCMC sampler

In the following, we describe a few experiments that were conducted to better understand the behavior of the MCMC sampler employed by SnappNet— in particular its efficiency at sampling from network space, and how this efficiency is affected by the priors on phylogenetic network and population sizes. The prior on phylogenetic networks is specified in terms of the birth-hybridization model by Zhang et al. [7].

8.1 Experiment with no data

8.1.1 Protocol

In the first experiment we assess whether the MCMC sampler employed by SnappNet can adequately sample from network space. We specify a posterior distribution over 5-taxon phylogenetic networks with high variance across multiple number of reticulations. We ran the MCMC sampler so that it sampled from a posterior distribution specified in terms of a birth-hybridization model prior, origin height prior and a null likelihood function (always returns zero regardless of the input data). We then compared the sampled networks with 5-taxon networks simulated directly from the birth-hybridization model. Theoretically we expect the distributions of sampled and simulated networks to match.

We studied three different cases of the birth-hybridization model prior, for each case we either specified a normal prior with mean 0.1 and standard deviation of 0.01 on the origin height or an exponential prior with mean 0.1 on the origin height (that is a total of six different scenarios): In the first case we used a birth-hybridization model with speciation rate 20 and hybridization rate 1 (mean number of reticulations close to zero). In the second case we used a birth-hybridization model with speciation rate 20 and hybridization rate 2 (mean number of reticulations close to one). In the third case we used a birth-hybridization model with speciation rate 20 and hybridization rate 3 (mean number of reticulations close to two). We only kept simulated networks with 5 leaves.

In each case we simulated 1000 networks directly from the birth-hybridization model and sampled 2,000,000 networks using the SnappNet sampler (burning half the chain and logging every 1000th sample thereafter). Note that it is possible to fix the birth and hybridization rates in the prior used by SnappNet by fixing corresponding values for parameters d and r. We used Tracer to assess convergence of the MCMC chain by visually inspecting the trace and computing the ESS (effective sample size). Thereafter we compared the simulated networks with sampled networks in terms of the number of reticulations, time until first reticulation, network height and network length.

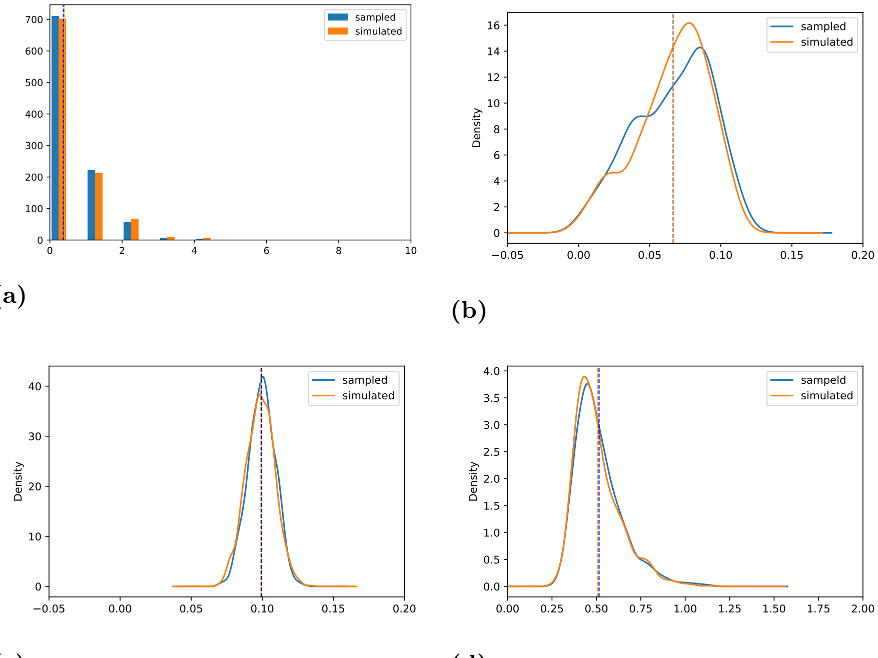

(a) **(b)** **(c)** **(d)**

Figure O. Birth-hybridisation model with speciation rate 20 and hybridisation rate 1 (mean number of reticulations close to zero) and a normal prior with mean 0.1 and standard deviation of 0.01 on the origin height. We plot the simulated networks (orange) against the sampled networks (blue) summarising the networks under: (a) Number of reticulations (b) Time until first reticulation (c) Height of the network (d) Length of the network.

8.1.2 Results

In the first experiment the sampler converged to the specified prior in all three cases (for both origin height priors) based on the computed summary statistics (see Figs O-Q and Figs R-T). The convergence of the sampler in all cases is a good indication that the implemented moves worked well enough. The ESS for the sampled networks given the normal prior on the network origin were: 1001 for the first case (mean number of reticulation close to zero); 844 for the second case (mean number of reticulations close to one); 1001 for the third case (mean number of reticulations close to two). The ESS for the sampled networks given the exponential prior on the network origin were: 872 for the first case; 955 for the second case; 838 for the third case.

Note that the normal and exponential priors on the origin height permit to describe different knowledge on the expected number of reticulations, see Figs Q(a) and T(a).

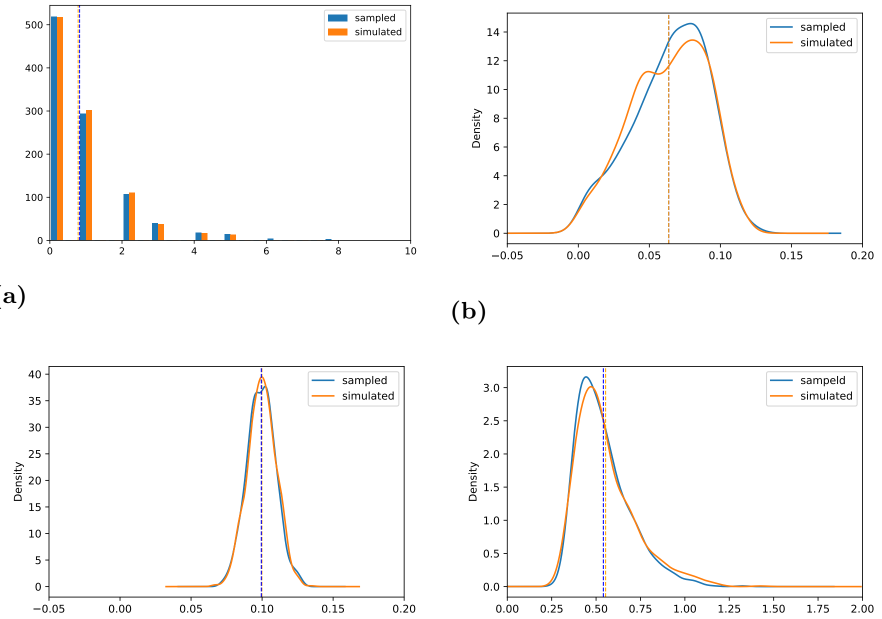

(a) (b) (c) (d)

Figure P. Birth-hybridisation model with speciation rate 20 and hybridisation rate 2 (mean number of reticulations close to one) and normal prior with mean 0.1 and standard deviation of 0.01 on the origin height. We plot the simulated networks (orange) against the sampled networks (blue) summarising the networks under: (a) Number of reticulations (b) Time until first reticulation (c) Height of the network (d) Length of the network.

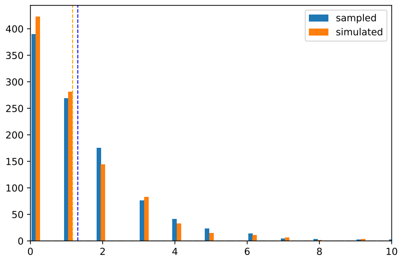

(a)

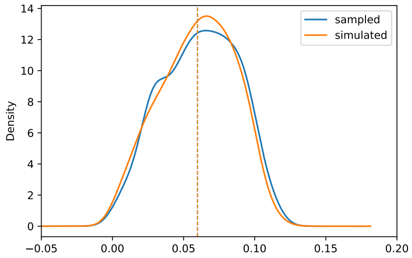

(b)

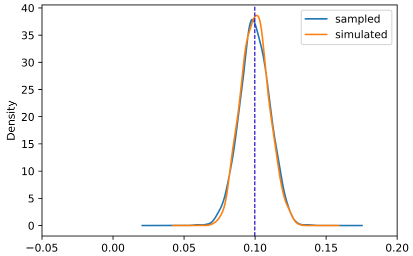

(c)

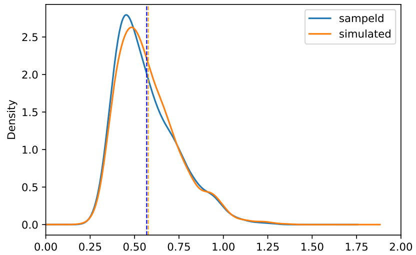

(d)

Figure Q. Birth-hybridisation model with speciation rate 20 and hybridisation rate 3 (mean number of reticulations close to two) and normal prior with mean 0.1 and standard deviation of 0.01 on the origin height. We plot the simulated networks (orange) against the sampled networks (blue) summarising the networks under: (a) Number of reticulations (b) Time until first reticulation (c) Height of the network (d) Length of the network.

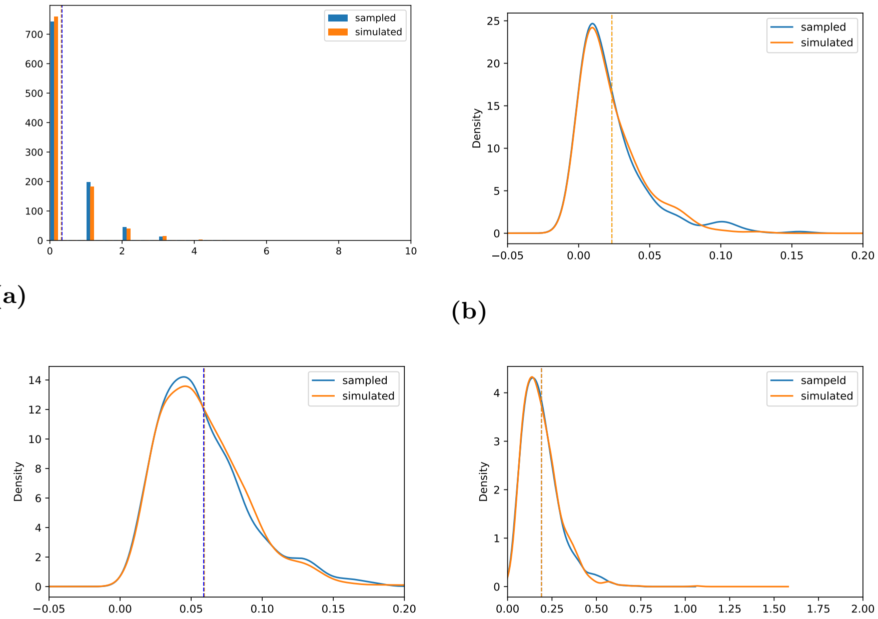

(a)

(b)

(c)

(d)

Figure R. Birth-hybridisation model with speciation rate 20 and hybridisation rate 1 (mean number of reticulations close to zero) and an exponential prior with mean 0.1 on the origin height. We plot the simulated networks (orange) against the sampled networks (blue) summarising the networks under: (a) Number of reticulations (b) Time until first reticulation (c) Height of the network (d) Length of the network.

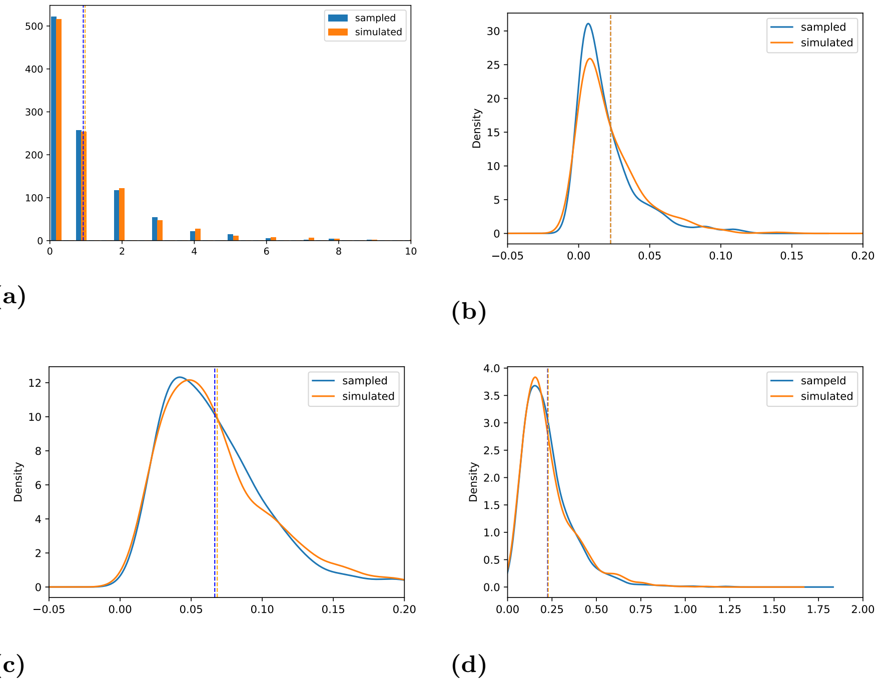

Figure S. Birth-hybridisation model with speciation rate 20 and hybridisation rate 2 (mean number of reticulations close to one) and an exponential prior with mean 0.1 on the origin height. We plot the simulated networks (orange) against the sampled networks (blue) summarising the networks under: (a) Number of reticulations (b) Time until first reticulation (c) Height of the network (d) Length of the network.

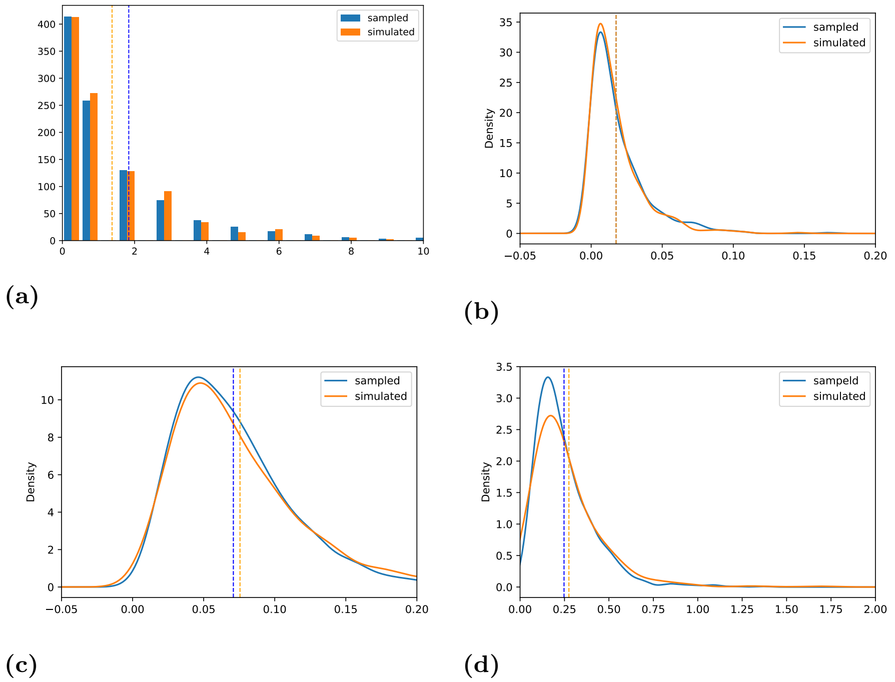

Figure T. Birth-hybridisation model with speciation rate 20 and hybridisation rate 3 (mean number of reticulations close to two) and an exponential prior with mean 0.1 on the origin height. We plot the simulated networks (orange) against the sampled networks (blue) summarising the networks under: (a) Number of reticulations (b) Time until first reticulation (c) Height of the network (d) Length of the network.

#Chain	Network prior	Mean reticulations	Pop size prior
1,2	BH(20,1)	0.371	$\Gamma(1, 200)$
3,4	BH(20,1)	0.371	$\Gamma(1, 20)$
5,6	BH(20,1)	0.371	$\Gamma(1, 1000)$
7,8	BH(20,1)	0.371	$\Gamma(1, 2000)$
9,10	BH(20,2)	0.861	$\Gamma(1, 200)$
11,12	BH(20,2)	0.861	$\Gamma(1, 20)$
13,14	BH(20,2)	0.861	$\Gamma(1, 1000)$
15,16	BH(20,2)	0.861	$\Gamma(1, 2000)$
17,18	BH(20,3)	2.265	$\Gamma(1, 200)$
19,20	BH(20,3)	2.265	$\Gamma(1, 20)$
21,22	BH(20,3)	2.265	$\Gamma(1, 1000)$
23,24	BH(20,3)	2.265	$\Gamma(1, 2000)$

Table G. BH(birth rate, hybridisation rate) refers to the birth-hybridisation process of Zhang et al. with the specified birth and hybridisation rates. For data simulated with network A, only chains 1,2,3,4,9,10,11,12,17,18,19,20 were run. We indicate the mean number of reticulation for the Birth-Hybridization model given an exponential prior with mean 0.1 on network origin. Note that we only used the exponential prior in the experiment in Section 8.2.

8.2 Experiments on 10,000 simulated sites

8.2.1 Protocol

In the second experiment we assess how population size priors and network priors influence SnappNet's inferences, in particular the rate of convergence and sampling efficiency of the MCMC sampler. Recall that the network prior specifies a hybridization rate, whereas the prior on population sizes affects the probability of coalescence, and therefore that of ILS. Thus, these two priors have an important role in determining the relative probability of hybridization and ILS as causes of incongruent (non-tree-like) signals in the data.

We simulated 10,000 SNPs for network A and network B under the multispecies network coalescent using SimSnappNet. For each of these two simulated SNP datasets, we ran 12 (for network A) or 24 (for network B) MCMC chains, for 500,000 iterations each. See Table G for details on the priors specified for each chain. In this experiment we only use the exponential prior with mean 0.1 on the network origin.

Briefly, as in the experiment of Sec. 8.1, we specified a network prior using the birth-hybridisation model of Zhang et al. [7]. Again, we fixed the birth rate to 20 for all MCMC chains and chose a hybridisation rate so that the mean number of reticulations is close to zero, one or two. Furthermore we specified either a 'correct' or 'incorrect' prior on population size ('correct' implies the mean of the prior distribution corresponds to the population size parameter used to simulate the SNP dataset). The 'correct' population size prior on each

branch was specified as $\Gamma(1, 200)$. The 'incorrect' population size prior on each branch was specified as $\Gamma(1, 20)$. For network B we considered two additional incorrect population size priors, namely $\Gamma(1, 1000)$ and $\Gamma(1, 2000)$. Note that the rest of the priors of the model used the default SnappNet settings. In order to assess convergence we ran two MCMC chains for each prior setting (as specified in Table G). We randomly drew initial networks and population sizes for each MCMC chain from the prior distribution. Also note that, here we do not impose any upper bound on the number of reticulations in the sampled networks.

8.2.2 Results for network A

We summarize results for data simulated under network A in Fig U (MCMC chains with correct population size priors) and Fig V (MCMC chains with incorrect population size priors). We also give detailed summary statistics in Table H (MCMC chains with correct population size priors) and Table I (MCMC chains with incorrect population size priors). We note that all chains with correct population size priors converged to the correct topology, network height and network length (see Figs U(c) and U(d)). We assume convergence for network topology since there was only one unique topology for each posterior distribution of the chains with correct population size priors. In each case the unique topology matched up with the topology of network A. Furthermore in Fig U(b) all chains have similar prior distributions. This could be due to the topology of network A that is very unlikely under all the specified birth-hybridization model priors (similar to sampling from a flat prior). We also note a much lower ESS under the model prior with reticulation mean close to zero (see ESS in Table H).

Chains with incorrect population size priors also converged to the correct topology. Similar to correct priors, there was only one unique topology for all chains. However the chains did not converge to the correct network height or network length. This is not unexpected since the length of a branch and its associated population size are correlated (see Bryant et al. [2] for more detail). Furthermore the ESS for chains with incorrect population size priors is significantly lower than chains with correct population size priors (see ESS in Table H and Table I). There is also a difference in ESS between chains with different topology priors. In this case ESS is highest when the mean number of reticulations on the network topology prior is close to one and lowest when mean number of reticulations for the network topology prior is close to two. This seems to suggest that specifying a prior with correct mean number of reticulations can improve sampling efficiency.

Table H. MCMC summary statistics for network A (correct population size priors)

**Posterior**	0	1	2
mean	-16124.9199	-16123.2496	-16123.8596
stdev	4.7795	4.9024	5.0388
median	-16124.601	-16122.7308	-16123.2257

95% HPD Interval	[-16134.1, -16116.4]	[-16133.4, -16114.9]	[-16133.0, -16114.7]
Auto-correlation time	4537.3247	1336.3834	1454.4489
Effective sample size	198.5752	674.2077	619.4786
**Network height**	0	1	2
mean	0.076	0.0769	0.0767
stdev	5.12E-03	4.09E-03	4.34E-03
median	0.0768	0.0772	0.0774
95% HPD Interval	[0.0647, 0.0854]	[0.0686, 0.0844]	[0.0682, 0.0847]
Auto-correlation time	10376.3331	3552.1422	3800.8964
Effective sample size	86.8322	253.6498	237.0493
**Network length**	0	1	2
mean	0.213	0.2144	0.2137
stdev	0.0124	9.93E-03	0.0107
variance	1.54E-04	9.87E-05	1.14E-04
95% HPD Interval	[0.1882, 0.2369]	[0.1967, 0.2357]	[0.1934, 0.2344]
Auto-correlation time	7533.4457	3113.3204	3818.75
Effective sample size	119.6	289.4016	235.9411

Table I. MCMC summary statistics for network A (incorrect priors)

**Posterior**	0	1	2
mean	-15953.476	-15917.2941	-15917.7676
stdev	5.1764	7.0277	6.7969
median	-15952.9488	-15916.1712	-15917.1603
95% HPD Interval	[-15962.4, -15943.6]	[-15932.0, -15905.4]	[-15930.3, -15905.5]
Auto-correlation time	2395.3008	2164.8158	3002.7331
Effective sample size	167.4111	185.2352	133.545
**Network height**	0	1	2
mean	0.0548	0.0588	0.047
stdev	9.30E-03	0.0121	0.0112
median	0.0548	0.06	0.0476
95% HPD Interval	[0.0394, 0.073]	[0.0383, 0.0791]	[0.025, 0.0662]
Auto-correlation time	16248.1423	1.00E+05	34725.0867
Effective sample size	24.6797	4.0042	11.5478
**Network length**	0	1	2
mean	0.1655	0.1783	0.156
stdev	0.0206	0.0252	0.0255
median	0.1649	0.1805	0.158
95% HPD Interval	[0.1264, 0.2024]	[0.1331, 0.2263]	[0.1064, 0.201]
Auto-correlation time	11802.0222	74214.3724	27882.5047
Effective sample size	33.9772	5.4033	14.3818

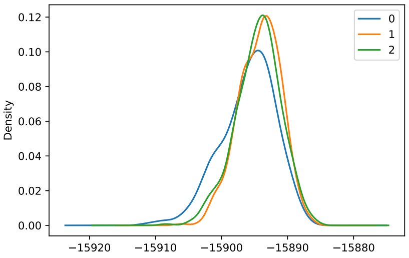

(a)

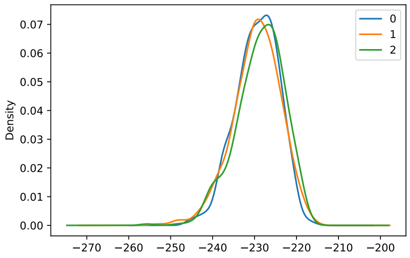

(b)

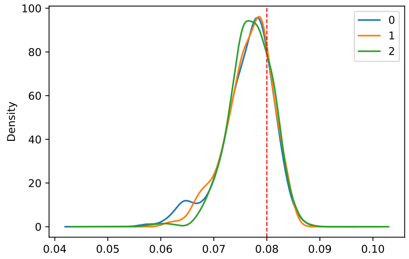

(c)

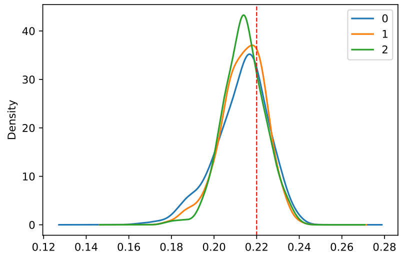

(d)

Figure U. Summary distributions of all chains with correct population size priors (chain numbers 1,2,9,10,17,18) given data simulated from network A. We summarize the MCMC chains by combining them, that is: Chains 1 and 2 are indicated by the blue line (mean reticulations close to zero); Chains 9 and 10 are indicated by the orange line (mean reticulations close to one); Chains 17 and 18 are indicated by the green line (mean reticulations close to two); We plot the following distributions (a) Likelihood (b) Prior (c) Network height (d) Network length. Note that network height and network length used to simulate data are indicated by red lines.

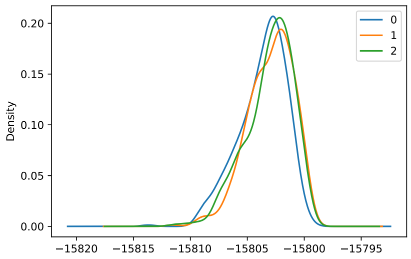

(a)

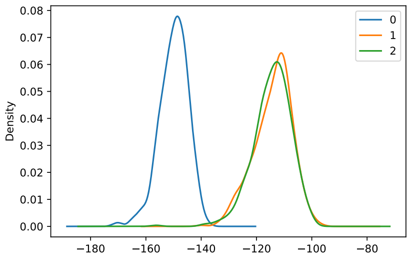

(b)

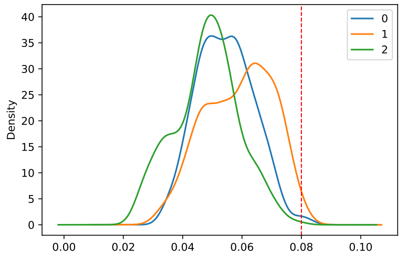

(c)

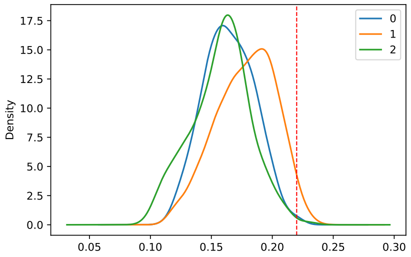

(d)

Figure V. Summary distributions of all chains with incorrect population size priors Gamma(1,20) (chain numbers 3,4,11,12,19,20) given data simulated from network A. We summarize the MCMC chains by combining them, that is: Chains 3 and 4 are indicated by the blue line (mean reticulations close to zero); Chains 11 and 12 are indicated by the orange line (mean reticulations close to one); Chains 19 and 20 are indicated by the green line (mean reticulations close to two); We plot the following distributions (a) Likelihood (b) Prior (c) Network height (d) Network length. Note that network height and network length used to simulate data are indicated by red lines.

8.2.3 Results for network B

We summarize results for data simulated under network B in Fig W (MCMC chains with correct population size priors), Fig X (MCMC chains with incorrect population size priors $\Gamma(1, 20)$), Fig Y (MCMC chains with incorrect population size priors $\Gamma(1, 1000)$) and Fig Z (MCMC chains with incorrect population size priors $\Gamma(1, 2000)$). We also give detailed summary statistics in Table J (MCMC chains with correct population size priors), Table K (MCMC chains with incorrect population size priors $\Gamma(1, 20)$), Table L (MCMC chains with incorrect population size priors $\Gamma(1, 1000)$) and Table M (MCMC chains with incorrect population size priors $\Gamma(1, 2000)$). We note that all chains with correct population size priors converged to the correct topology (posterior distribution contained only one network topology), network height and network length (see Figs W(c) and W(d)). Chains with incorrect population size priors also converged to the correct topology in most cases except for two cases: {BH(20,1),$\Gamma(1, 1000)$} and {BH(20,1),$\Gamma(1, 2000)$}. Therefore we were able to recover the correct topology 83.33% of the time. This is consistent with results in the simulation study of the main text. There is also a difference in ESS between chains with different topology priors. However in this case it is not clear how the prior affects the sampling efficiency (see ESS of Posterior distribution in Table J, Table K, Table L and Table M).

Table J. MCMC summary statistics for Network B (correct population size priors)

**Posterior**	0	1	2
mean	-16693.3667	-16692.247	-16690.8344
stdev	10.9267	11.2221	7.5266
median	-16692.4881	-16691.493	-16690.2388
95% HPD Interval	[-16706., -16678.2155]	[-16705.0995, -1667389	[-16705., -16676.7232]
Auto-correlation time	1000	1032	1138.8635
Effective sample size	501	489	439.9122
**Network height**	0	1	2
mean	0.0793	0.0796	0.0797
stdev	4.08E-03	3.71E-03	3.37E-03
median	0.0799	0.0797	0.08
95% HPD Interval	[0.0714, 0.086]	[0.072, 0.0858]	[0.0729, 0.0852]
Auto-correlation time	4047.2001	2842.2081	2047.1454
Effective sample size	123.7893	176.2714	244.731
**Network length**	0	1	2
mean	0.2421	0.2412	0.2412
stdev	0.0106	0.0106	9.82E-03
median	0.2431	0.2417	0.2418
95% HPD Interval	[0.2223, 0.2621]	[0.2229, 0.2625]	[0.2193, 0.2588]
Auto-correlation time	3262.683	2788.3608	1948.3473
Effective sample size	153.5546	179.6755	257.141

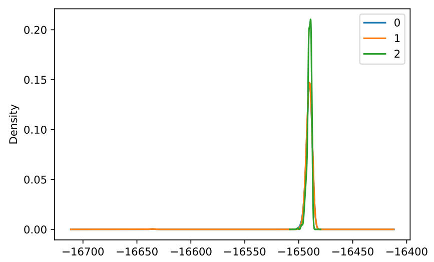

(a)

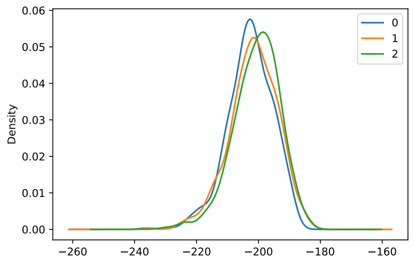

(b)

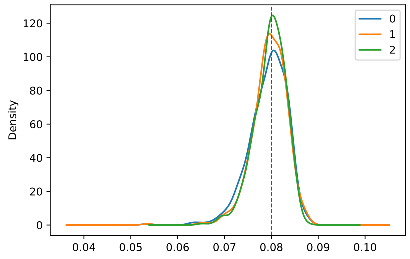

(c)

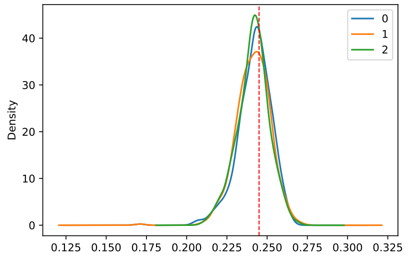

(d)

Figure W. Summary distributions of all chains with correct population size priors (chain numbers 1,2,9,10,17,18 given data simulated under network B. We summarize the MCMC chains by combining them, that is: Chains 1 and 2 are indicated by the blue line (mean reticulations close to zero); Chains 9 and 10 are indicated by the orange line (mean reticulations close to one); Chains 17 and 18 are indicated by the green line (mean reticulations close to two); We plot the following distributions (a) Likelihood (b) Prior (c) Network height (d) Network length. Note that network height and network length used to simulate data are indicated by red lines.

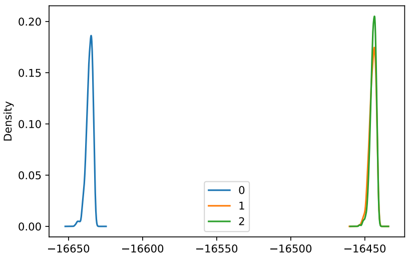

(a)

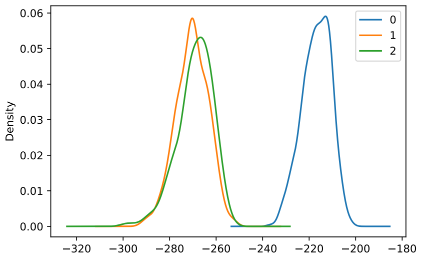

(b)

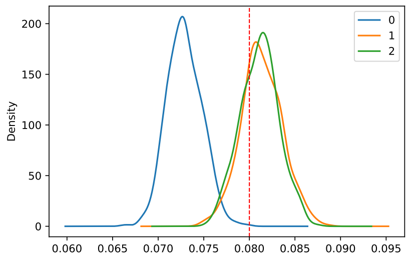

(c)

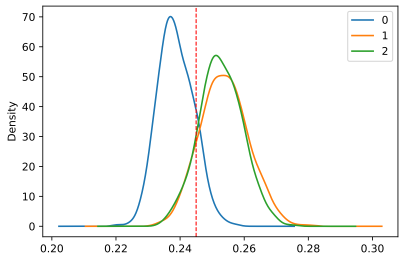

(d)

Figure X. Summary distributions of all chains with incorrect population size priors (chain numbers 3,4,7,8,11,12) given data simulated from network B. We summarize the MCMC chains by combining them, that is: Chains 3 and 4 are indicated by blue line (mean reticulations close to zero); Chains 7 and 8 are indicated by orange line (mean reticulations close to one); Chains 11 and 12 are indicated by green line (mean reticulations close to two); We plot the following distributions (a) Likelihood (b) Prior (c) Network height (d) Network length. Note that network height and network length used to simulate data are indicated by red lines.

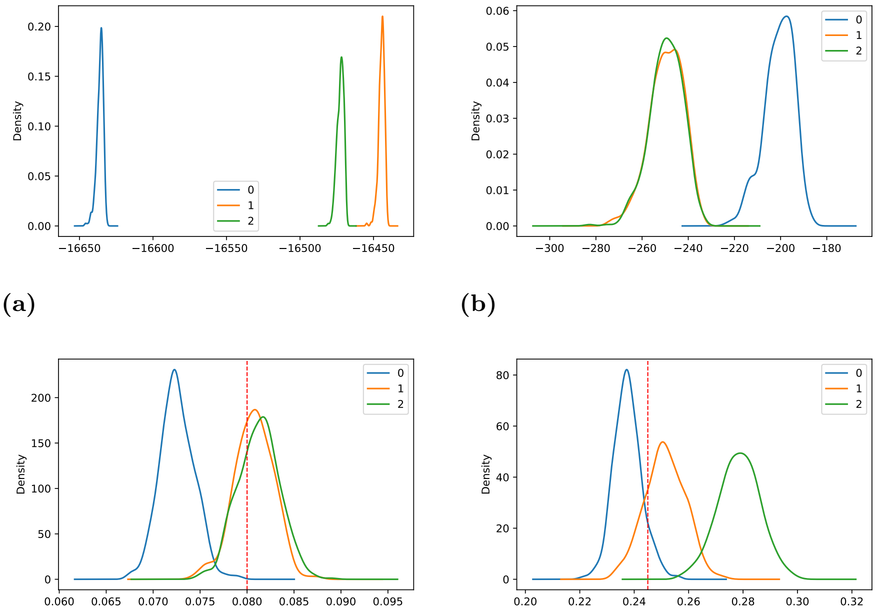

(a) **(b)** **(c)** **(d)**

Figure Y. In this figure we plot summary distributions of all chains with incorrect population size priors Gamma(1,20) (chain numbers 5,6,13,14,21,22) given data simulated from Network B. We summarize the MCMC chains by combining them, that is: Chains 5 and 6 are indicated by blue line (mean reticulations close to zero); Chains 13 and 14 are indicated by orange line (mean reticulations close to one); Chains 21 and 22 are indicated by green line (mean reticulations close to two); We plot the following distributions (a) Likelihood (b) Prior (c) Network height (d) Network length. Note that network height and network length used to simulate data are indicated by red lines.

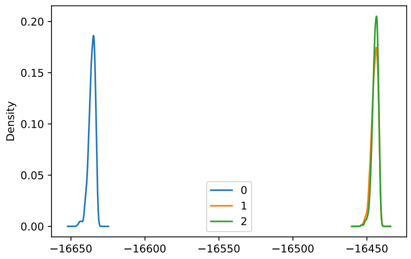

(a)

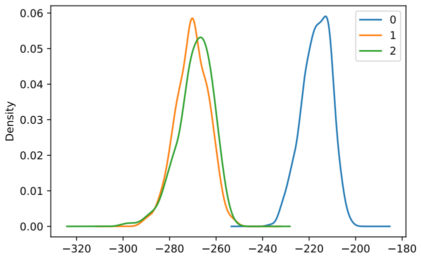

(b)

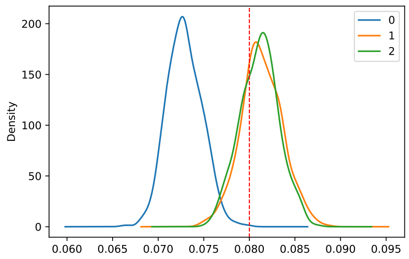

(c)

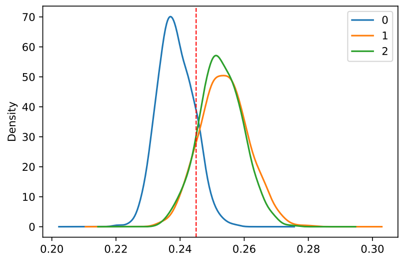

(d)

Figure Z. In this figure we plot summary distributions of all chains with incorrect population size priors (chain numbers 7,8,15,16,23,24) given data simulated from network B. We summarize the MCMC chains by combining them, that is: Chain 7 and 8 are indicated by blue line (mean reticulations close to zero); Chain 15 and 16 is indicated by orange line (mean reticulations close to one); Chain 23 and 24 are indicated by green line (mean reticulations close to two); We plot the following distributions (a) Likelihood (b) Prior (c) Network height (d) Network length. Note that network height and network length used to simulate data are indicated by red lines.

Table K. MCMC summary statistics for Network B (incorrect population size priors Gamma(1,20))

**Posterior**	0	1	2
mean	-16632.0842	-16630.1919	-16629.0074
stdev	8.0517	8.0343	8.3013
median	-16631.0958	-16629.7991	-16627.8702
95% HPD Interval	[-16647.6, -16617.2]	[-16646.3, -16615.2]	[-16644.3, -16613.8]
Auto-correlation time	3035.0395	2302.8325	1819.3308
Effective sample size	132.1235	174.1334	220.4107
**Network height**	0	1	2
mean	0.0639	0.0568	0.0578
stdev	0.011	0.0119	0.0117
median	0.0645	0.0558	0.0571
95% HPD Interval	[0.044, 0.0838]	[0.039, 0.083]	[0.0392, 0.0789]
Auto-correlation time	25480.1793	59294.2576	31555.2738
Effective sample size	15.7377	6.7629	12.7079
**Network length**	0	1	2
mean	0.1958	0.1826	0.1831
stdev	0.024	0.0253	0.0255
median	0.196	0.1836	0.1827
95% HPD Interval	[0.1543, 0.2412]	[0.1398, 0.2311]	[0.1378, 0.2273]
Auto-correlation time	19208.3139	36324.2679	23849.9552
Effective sample size	20.8764	11.0395	16.8134

Table L. MCMC summary statistics for Network B (incorrect population size priors Gamma(1,1000))

**Posterior**	0	1	2
mean	-16836.9572	-16693.995	-16722.2755
stdev	6.9406	7.7519	7.5566
median	-16836.2496	-16693.5673	-16722.1075
95% HPD Interval	[-16850.7, -16824.8]	[-16709.2, -16679.9]	[-16738.3, -16709.7]
auto-correlation time (ACT)	1665.166	1018.3201	1230.9875
effective sample size (ESS)	240.8168	393.7858	325.7547
**Network height**	0	1	2
mean	0.0726	0.0807	0.0811
stdev	1.88E-03	2.13E-03	2.20E-03
median	0.0724	0.0808	0.0811
95% HPD Interval	[0.0695, 0.0764]	[0.0768, 0.0847]	[0.0774, 0.0854]
auto-correlation time (ACT)	1000	1199.9843	1160.6579
effective sample size (ESS)	401	334.171	345.4937
**Network length**	0	1	2
mean	0.2375	0.2511	0.2781
stdev	5.38E-03	7.50E-03	7.67E-03
median	0.2371	0.2509	0.2784
95% HPD Interval	[0.228, 0.2492]	[0.2374, 0.2664]	[0.2633, 0.2928]

auto-correlation time (ACT)	1000	1221.2116	1413.5422
effective sample size (ESS)	401	328.3624	283.6845

Table M. MCMC summary statistics for Network B (incorrect population size priors Gamma(1,2000))

**Posterior**	0	1	2
mean	-16852.5586	-16715.134	-16713.4537
stdev	6.4594	7.6484	7.6353
95% HPD Interval	[-16864.9, -16841.0]	[-16731.4, -16701.6]	[-16727.7, -16698.8]
auto-correlation time (ACT)	1216.2537	1762.3287	1120.84
effective sample size (ESS)	329.7009	227.5398	357.7674
**Network height**	0	1	2
mean	0.0729	0.0813	0.0811
stdev	1.92E-03	2.25E-03	2.10E-03
95% HPD Interval	[0.0697, 0.0769]	[0.0767, 0.0855]	[0.0771, 0.0852]
auto-correlation time (ACT)	1455.1062	1073.7888	1425.0583
effective sample size (ESS)	275.5813	373.444	281.392
**Network length**	0	1	2
mean	0.2386	0.2539	0.2524
stdev	5.61E-03	7.39E-03	6.74E-03
variance	3.14E-05	5.46E-05	4.55E-05
95% HPD Interval	[0.2284, 0.2493]	[0.239, 0.2668]	[0.239, 0.2653]
auto-correlation time (ACT)	1519.2302	1462.2703	1020.1866
effective sample size (ESS)	263.9495	274.2311	393.0654

8.2.4 Operator acceptance rates

To better understand the behavior of the MCMC sampler, we inspect the acceptance rates for the 5 operators acting on the network topology (*AddReticulation, DeleteReticulation, FlipReticulation RelocateBranch, RelocateBranchNarrow*), the 4 operators updating branch lengths (*NodeSlider, NodeUniform, NetworkMultiplier, OriginMultiplier*) and the 2 operators updating population sizes (*ChangeGamma, ChangeAllGamma*).

We summarize the acceptance rates for network B in Table N, Table O and Table P. Each table focuses on a different population size prior, while averaging across the topology priors.

We observe that MCMC moves that update topology have a much lower acceptance rate than MCMC moves that update branch lengths and population sizes. *FlipReticulation* moves, which flip the direction of a reticulation branch, are the least likely to be accepted. There is no clear difference in the acceptance rates between different population size priors. More work is needed to determine what the proposal weights should be in order to optimally sample from the posterior distribution.

Table N. MCMC acceptance rates for Network B (correct population size priors).

Id	Pr_accept	Pr_proposed	Pr_proposed	Pr_accept
**Topology moves**				
AddReticulation	1.43E-04		2.32E-02	3.31E-06
DeleteReticulation	4.55E-05		2.32E-02	1.06E-06
FlipReticulation	8.05E-06		2.35E-02	1.89E-07
RelocateBranch	3.07E-02		2.34E-02	7.20E-04
RelocateBranchNarrow	1.81E-03		2.33E-02	4.21E-05
**Branch length**				
NodeSlider	5.29E-01		2.32E-02	1.23E-02
NodeUniform	2.73E-01		2.32E-02	6.33E-03
NetworkMultiplier	2.92E-01		1.15E-02	3.36E-03
OriginMultiplier	7.50E-01		1.18E-02	8.86E-03
**Population size**				
ChangeGamma	3.16E-01		3.49E-01	1.10E-01
ChangeAllGamma	2.80E-01		3.48E-01	9.75E-02

Table O. MCMC acceptance rates for Network B (incorrect population size priors $\Gamma(1, 1000)$).

Id	Pr_accept	Pr_proposed	Pr_proposed	Pr_accept
**Topology moves**				
AddReticulation	1.52E-04		2.33E-02	3.55E-06
DeleteReticulation	6.72E-05		2.32E-02	1.56E-06
FlipReticulation	1.44E-05		2.33E-02	3.33E-07

RelocateBranch	2.59E-02		2.33E-02	6.02E-04
RelocateBranchNarrow	6.01E-04		2.34E-02	1.41E-05
**Branch length**				
NodeSlider	5.20E-01		2.33E-02	1.21E-02
NodeUniform	2.68E-01		2.32E-02	6.22E-03
NetworkMultiplier	2.58E-01		1.15E-02	2.97E-03
OriginMultiplier	7.42E-01		1.17E-02	8.67E-03
**Population size**				
ChangeGamma	3.36E-01		3.49E-01	1.17E-01
ChangeAllGamma	3.15E-01		3.49E-01	1.10E-01

Table P. MCMC acceptance rates for Network B (incorrect population size priors $\Gamma(1, 2000)$).

Id	Pr_accept	Pr_proposed	Pr_proposed	Pr_accept
**Topology moves**				
AddReticulation	1.14E-04		2.32E-02	2.64E-06
DeleteReticulation	3.25E-05		2.32E-02	7.54E-07
FlipReticulation	2.43E-05		2.33E-02	5.68E-07
RelocateBranch	2.71E-02		2.33E-02	6.31E-04
RelocateBranchNarrow	5.49E-04		2.33E-02	1.28E-05
**Branch length**				
NodeSlider	5.11E-01		2.33E-02	1.19E-02
NodeUniform	2.53E-01		2.32E-02	5.86E-03
NetworkMultiplier	2.65E-01		1.15E-02	3.04E-03
OriginMultiplier	7.46E-01		1.18E-02	8.77E-03
**Population size**				
ChangeGamma	3.69E-01		3.49E-01	1.29E-01
ChangeAllGamma	3.80E-01		3.49E-01	1.32E-01

References

[1] Zhu J, Wen D, Yu Y, Meudt HM, Nakhleh L. Bayesian inference of phylogenetic networks from bi-allelic genetic markers. PLoS computational biology. 2018;14(1):e1005932.

[2] Bryant D, Bouckaert R, Felsenstein J, Rosenberg NA, RoyChoudhury A. Inferring species trees directly from biallelic genetic markers: bypassing gene trees in a full coalescent analysis. Molecular biology and evolution. 2012;29(8):1917–1932.

[3] Bryant D, RoyChoudhury A, Bouckaert R, Felsenstein J, Rosenberg N. Exact coalescent likelihoods for unlinked markers in finite-sites mutation models. arXiv preprint arXiv:11093525. 2011;.

[4] Griffiths RC, Tavare S. Computational methods for the coalescent. IMA Volumes in Mathematics and its Applications. 1997;87:165–182.

[5] Huson DH, Rupp R, Scornavacca C. Phylogenetic networks: concepts, algorithms and applications. Cambridge University Press; 2010.

[6] Gambette P, Berry V, Paul C. The structure of level-k phylogenetic networks. In: Annual Symposium on Combinatorial Pattern Matching. Springer; 2009. p. 289–300.

[7] Zhang C, Ogilvie HA, Drummond AJ, Stadler T. Bayesian inference of species networks from multilocus sequence data. Molecular biology and evolution. 2017;35(2):504–517.

[8] Wang W, Mauleon R, Hu Z, Chebotarov D, Tai S, Wu Z, et al. Genomic variation in 3,010 diverse accessions of Asian cultivated rice. Nature. 2018;557(7703):43–49.
