# Supplementary material for: On the inference of complex phylogenetic networks by Markov Chain Monte-Carlo
Source: PLoS Comput Biol. 2021 Sep 3;17(9):e1008380. doi: 10.1371/journal.pcbi.1008380 (PMC8445492; doi:10.1371/journal.pcbi.1008380)
Supplement: Attachment — Submitted filename: Answers.pdf [file pcbi.1008380.s002.pdf]

Response to reviewer comments

We thank the editor and reviewers for the many helpful and constructive comments.

We have made very substantial changes to the manuscript, most notably we have re-run the analyses on rice data using new data sets that were constructed thanks to the help of João Santos and Wensheng Wang. We were fortunate to launch some of these new analyses last October, which gave us the time to obtain satisfying results with the MCMC approach implemented in SnappNet (unlike in the previous version). Marnus Stoltz helped us with the new analyses presented in Section 8 of the Supplement. João Santos, Wensheng Wang and Marnus Stoltz are now added to the author list.

We have thoroughly revised the entire manuscript, including the English, as this was probably affecting its overall clarity. All changes to the manuscript and to the supplementary material (only section titles are colored in latter when the whole section is new) are highlighted in color, giving the opportunity to the editor and reviewers to appreciate the extensiveness of this revision.

Yours sincerely,

The authors

Deputy Editor and Associate Editor main comment

Particular attention should be paid to overhauling the "real data" analysis part of the manuscript (the reviewers were quite disappointed by the current one), and to improving the depth and clarity (although opinions differed there) of the methodological exposition.

• We completely rewrote the part on real data, which now presents an entirely new Bayesian analysis of rice data. We were indeed quite unsatisfied with the difficulty of reaching convergence on our old data sets. Because of this, at the time of the first submission (October 2020) we launched MCMC analyses of 3 new data sets, whose main difference with the old data sets is that they include 3 new taxa representing 3 sub-populations of wild rice.

• The increased taxon sampling — combined with the fact that some of these analyses have been running uninterrupted until now (about 7 months) — allowed us to reach results that we consider satisfying, especially because they converged to topologically identical results (except in one case). This new Bayesian analysis is now described and discussed in new text appearing at lines 470, 687, and 848.

• We also did our best to address all the points where our methodological exposition needed to be explained in more detail. (Cf. all the new text that has been added to the Material and

methods).

Review 1

The authors presented an extremely well-written manuscript with the extension of SNAPP to networks (SnappNet) which is similar in principle to the work on Zhu et al (2018) but with two key differences: the way in which the likelihood is computed and the priors. These two differences have a huge impact on the method's computational speed and convergence.

I want to thank the authors for the high quality writing and figures. It was a joy to read it!

• We thank the reviewer for the positive comment.

Major comments:

1. I would appreciate a bit more details or examples (maybe figures?) to better explain two concepts: ”population interfaces” and ”incomparable population interfaces”. The whole section on partial likelihoods relies on these two concepts and I found them very very hard to understand (perhaps given my inexperience with Snapp to begin with). For readers like me that are not familiar with these concepts, a bit more explanation will make this section much easier to digest

   • We've added a new figure (Fig. 2) that clarifies those concepts (and more). We have also modified the way incomparable population interfaces are introduced, which was indeed a bit convoluted in the previous text. The definition of incomparable interfaces is now given early, at line 215.

   • Note that the use of the word "incomparable" is only necessary to ensure the correctness of our rules. However **all** vectors of population interfaces generated by our algorithm consist of incomparable population interfaces, so the distinction between incomparable and comparable population interfaces is not really important to understand our algorithm. We now clarify this in a new paragraph starting at line 267.

2. Did you make experiments on how the prior affects convergence and computational speed? Given that the prior is an important difference with the work in Zhu et al (and that we know that it has some weight on the mixing of the chain), it would be interesting to compare how the network priors behave in terms of convergence and speed, not just accuracy

   • First, please note that our experiments on SnappNet's speed only focus on a single likelihood calculation, and therefore exclude the effect of the prior on the overall speed of the MCMC. This is now clearly stated at line 496. As a consequence, the superior efficiency that SnappNet shows in our experiments is definitely only due to its new likelihood algorithm and not to the different prior.

   • We did some experiments with `MCMC_BiMarkers` prior. The authors kindly provided us a jar file, that enabled us to run `MCMC_BiMarkers` without data, in order to sample from the prior. We let `MCMC_BiMarkers` run for 100 million steps, considering a 5 tips-network. Surprisingly, the ESS was very low: we obtained an ESS of 25. We repeated the experiment several times, and we could not obtain larger ESS values.

   • Separately we have looked into the effects of the new prior on convergence and speed. Many new results on the effects of the new prior on convergence are now reported in Section 8 of

the Supplementary Material. In these tests, we did not see any noticeable difference in the rate of convergence of topologies between the different priors.

3. It seems that the tests on the accuracy of the numerical parameters is done only on Network A (unless I missed this for Network B and C). I think that testing the accuracy of estimated branch lengths and network height is very important for complex networks like B and C, so I wish something could be said about this.

   • We chose to focus on network A for consistency and ease of comparison with the `MCMC_BiMarkers` paper.

   • Nevertheless, the tests that the reviewer suggests are indeed interesting. In the Supplementary Material, we have now added the accuracies of estimated branch lengths and population sizes for network B (cf. Figures 4, 5, 6 in Supplementary Material). Network height and network length can easily be deduced from these tables. Since we considered only one lineage per species in our previous experiments on network B (in the manuscript, line 501), we had to run new analyses with two lineages per species. (Otherwise, with only one lineage per species, some population sizes would be unidentifiable.) We considered only 10,000 sites because of the limited time for the revision. At the time of the submission of this revision, SnappNet has achieved an ESS of 200 for 14 replicates out of 20, and recovered the topology of Network B for 12 replicates out of these 14. As a result, our accuracies are based on the 12 replicates (cf. our plots in Supplementary Material).

   • We have also placed in Supplementary Material the accuracy of branch length estimates for network C (cf. Figure 7 in Supplementary Material). We considered the following experiment of the manuscript: 1 lineage in species A, D and O, and 4 lineages in species B and C. Our accuracies are based on analyses that recovered network C with ESS>100 as a criterion. We do not show the accuracy of population size estimates since population sizes for a few external branches are not identifiable in this setting (those incident with A, D and O). We could not run new analyses because of the time limit for the revision.

   • In the main manuscript, we are now referring to the additional Figures below line 555.

4. In addition, for complex networks it seems that the number of hybridizations was bounded at the true number of hybridizations which is a good initial test, but I would be interested to know how the method performs when the prior is more lax and allows for more complex networks. What networks do you end up visiting frequently if the prior allows for more hybridizations than the true number? I know that this type of tests could easily get out of hand, but doing one or two simulations in which the prior is not bounding the number of hybridizations could provide information on accuracy and convergence under more realistic settings. In fact, perhaps this could be one of the things happening on the real data, that the number of hybridizations is not correctly guessed for the prior.

   • We generated 3 new data sets from network B, with 10K Sites, and 2 lineages per species, and ran SnappNet with no bound on the number of reticulations. On the 3 sets, the posterior probability of network B was 100%. We obtained an ESS of 294.7, 364.6 and 336.4 for the 3 sets, after 1 600 000 steps. Note that these results are much less extensive than the ones in the paper (3 replicates instead of 20, and no consideration of "incorrect" priors), which is why we prefer to leave in the paper the results with the prior bound to 3 reticulations.

• On network C, we generated one dataset with 4 lineages in hybrid species B and C, and one lineage in species O, A and D. We considered 10,000 sites. After almost two months of calculations, SnappNet sampled 493 000 networks when it was run without any bound on the number of reticulations. On the same dataset, 1 797 000 iterations have been done with a number of reticulations bounded by 2. In both cases, SnappNet did not sample the true network C. This is not so surprising since results on Net C in the main manuscript are based on several months of calculations. Besides, in both cases, sampled networks always have 2 reticulations (after having discarded the burn-in). So, even when the number of reticulations is unbounded, SnappNet does not sample networks with too many reticulations. It must be due to the fact that we considered a larger number of sites (10,000). With only a few sites (ie 1,000), the result would have been different: we would end up with visiting more complex networks since, in this case, the prior has more weight as compared to the likelihood.

• The experiments that were conducted to better understand the behavior of the MCMC sampler employed by SnappNet –reported in Sec. 8 of the Supplement– confirm that, even when the the number of reticulations is unbounded, SnappNet efficiently samples from the network space.

5. It seems that Network C(3) and C(4) were only used to test CPU time and memory, which is indeed important, but if the algorithms were run anyway, it would be nice to see the accuracy of the method for these more complex networks

   • As we now state in the Materials and Methods, right where they are introduced (line 411), these networks represent a huge computational challenge for inference tools, so they were only used to evaluate the time and memory needed for a single likelihood calculation. This is also stated in the Results at line 493 and under "CPU time and required memory" (line 656). Since the MCMC was not run on these networks, we do not have information on the accuracy of the method for C(3) and C(4). We hope to be able to perform such tests in a future paper.

6. The real data analysis was the only disappointing part of the manuscript. The fact that the algorithm could not search adequately the space of networks and that 10 networks had to be arbitrarily chosen for comparison was very disappointing. It was also not explained what criteria was used to choose the 10 networks? I understand that real data will never behave like simulated data, but if the current algorithm is not applicable to this dataset (that appears to be quite similar, at least in terms of sample sizes to the simulated data), which real data could be used for this method? Also, do the authors have a sense for what made the real data so difficult to analyze? Because the sample size looks quite manageable (12,000 sites and 4 taxa). I think that a thorough investigation into why this dataset could not be analyzed with this method would provide users with more information regarding the limitations of the method

   • First, we have to mention that it is challenging to analyze rice data since there are many genetic exchanges in various directions, as suggested by many genetic studies. Thus, rice appears as a chance and a challenge for testing methods aiming to tackle phylogenetic resolution within a hybrid swarm.

   • In the new version of the manuscript, we have totally rewritten the different sections on real data (see the texts starting at lines 470, 687, 848 in the manuscript). Since we suspected that

the difficulty in the analysis of the rice data was partly due to insufficient taxon sampling, we decided to include data sampled in wild rices, while reducing the number of lineages per subpopulation. We now study 3 new data sets that include rice cultivars and also wild forms. Our data sets span the four main rice subpopulations of cultivars (Indica, Japonica, cAus and cBasmati), as well as the different types of wild rice *O. rufipogon* likely involved in the origin of cultivated rice.

• For this new study, the sample size is 7 subpopulations and 12 000 sites. Besides, we considered different numbers of varieties per subpopulation. We analyzed the 3 data sets with SnappNet in a Bayesian setting, and did not consider a penalized likelihood framework. The MCMC obtained results that we consider satisfactory, especially because they were consistent across different data sets. We refer to the result section starting on line 687 for more details. Note also that a few experiments were launched last October (e.g. data set 3), since we were not satisfied with the difficulty of reaching MCMC convergence on our old data sets.

• On these new data sets, SnappNet proves very efficient in resolving the three main phylogenetic pillars of current diversity in Asian rice (Civan et al 2015, Civan and Brown 2018) and revealing a hybrid origin for the iconic varietal group cBasmati (Santos et al 2019, Civan et al 2019). The various data sets treated here suggest a contribution of Japonica cultivars at a high level, between 0.6 and 0.85. We refer also to the new discussion on line 848.

Minor comments:

1. Line 62: "networks that can lead to observe such trees" sounds like it is referring to the concept of displayed tree which I think is not accurate in this section related to likelihood methods

   • Indeed that was potentially misleading, so we changed that sentence (line 53).

2. Line 228: I would add a "in this case" to the sentence of "Note that $N_{\underline{x}}$ is not random" because it is not random in general, right?

   • Yes, that's correct! Changed (now line 224).

3. Line 397: how are the "starting points" for the MCMC chosen?

   • The starting point was a tree for consistency with the Snapp and SpeciesNetwork software packages. None of these starting trees were subtrees of the correct network topology. We have made sure that all new analyses that we report now use a network randomly drawn from the prior distribution based on the birth-hybridization model by Zhang et al.

4. Line 427: why not say "estimated posterior probability" instead of "ratio", am I missing something?

   • This was indeed confusing. The new text starting at line 457 now says: "The posterior probability of the true network topology was then estimated by taking the proportion of sampled network topologies matching the true topology."

5. Lines 450-463: It is unclear the difference between the two optimizations. In the first case, you mention that only 9 operators were used (and ignored the 7 related to network updates) but in the second case you mention that the network topology was fixed. Isn't that the same thing as the first optimization?

• This part of the text has been removed since in this new version, we are using SnappNet exclusively in a Bayesian setting.

• In the previous version, we used two optimizations to maximize the likelihood. The first one consisted in accepting new parameter values, only when the likelihood increases. The second one consisted in accepting new parameter values using a MCMC algorithm (only the network topology was not allowed to change). This way, parameter values that led to a decrease of the likelihood could be sometimes accepted, and it enabled us to explore a different landscape. At the end, the highest likelihood among the two optimizations was retained.

6. Lines 471-473: I don't understand the evaluation criterion, can you clarify?

   • Indeed this was not well written. We want to evaluate the efficiency of calculating the likelihood here. We have rewritten this sentence (now at line 493).

7. Line 502: In the cases where a network with only 1 reticulation was recovered, was this at least a subnetwork of the true network?

   • This is now line 530 in the revised manuscript. This line refers to the 1-reticulation networks that `MCMC_BiMarkers` inferred for 1,000 sites simulated from network B (which has 2 reticulations). Recall that we did not re-run `MCMC_BiMarkers` here, but that our comparisons with `MCMC_BiMarkers` for networks A and B are based on the results published in Zhu et al. (Plos Comput Biol 2017). This was stated in the paragraph starting at line 402, but we have added a reminder of this at line 519.

   • As a consequence, we are unable to say if the 1-reticulation networks inferred by `MCMC_BiMarkers` are subnetworks of network B. One thing we can say with certainty is that `MCMC_BiMarkers` inferred multiple networks here (see the error bars in the bottom left panel of Fig. 5 of their paper). Moreover, at page 14 of Zhu et al. the authors wrote: "when the method sampled a tree, that tree did not exactly match the backbone tree in this case, but was close to it", which appears to mean that the inferred phylogenies were not always subnetworks of network B.

8. Table 1: I was intrigued that on the third row (less ILS) the accuracy in Network A decreased from 100% to 80% as you increased more sites. Do the authors have an idea why this could be happening?

   • This is indeed very interesting and there has been some debate among us about this. Note that this degradation of performance is observed for both incorrect priors (although for the prior $\beta = 1000$ this is much less remarkable). One possible explanation—which is hinted in our text—derives from the observation that one effect the incorrect priors is the introduction of supplementary edges in the inferred network. The reason why this happens is intuitive: the incorrect priors considered here (especially the one with $\beta = 2000$) underestimate the probability of ILS. This causes a tendency to favor hybridization over ILS as an explanation for incongruent (non-tree-like) signals in the data. This phenomenon will increase with the number of sites, because the signal for incongruence becomes stronger. It is thus to be expected that more sites will lead more often to overly complex networks. This is quite evident in the top row of Figure 9 (network A) for both incorrect priors.

9. Table 2: When the number of lineages are varied for B and C, you choose the same number of lineages for both, right? Either B and C have only one lineage, or they both have 4, correct?

• Yes. We clarified this in the caption of both Table 2 and Table 3. Also note that the main text erroneously said “C and D” (it was at line 593). Now this is corrected (line 621).

10. Table 7: there is a missing reference to a Figure

    • This comment does not apply anymore as all the tables about the 10 network analysis have been replaced by the results obtained with the new Bayesian analysis.

11. Figure 13: what do the authors refer to ”Network length”. I am sure this most be defined in the text, but I did not find it

    • It is defined in the Results (line 553), but indeed mentioning this in the Materials and Methods is a good idea, which we now do at line 466.

12. Figure 14 (and others): I would repeat the caption instead of writing ”Same as Figure 10” for readability

    • We expanded the captions of this and the other three figures, as requested. Since they were taking too much space in the main paper, we moved all these figures to the Supplement.

Review 2

Rabier et al. have developed an elegent and potentially revolutionary algorithm to compute the likelihood of biallelic data given a species network. Not only does their manuscript present the algorithm in sufficient clarity and detail, but several analyses of real and simulated data sets demonstrates its computational performance and general utility of biallelic inference of species networks.

I only have a few comments regarding the manuscript.

1. How was the correctness of the likelihood implementation validated? It would make sense to compare the likelihood computed by the algorithm of Zhu et al. with SnappNet for a random species network, disagreement would suggest an error in one or both implementations. Or it could possibly be computed manually for a simple network and compared with either implementation.

   • We did check the correctness of our likelihood calculations against `MCMC_BiMarkers` early on while developing SnappNet. At that time, the code of `MCMC_BiMarkers` was available online, so we modified it to print out a few likelihoods. The numerical values that we obtained were always nearly identical, with discrepancies consistent with rounding errors. As an example of this, Tables 1 and 2 below give the likelihoods that we obtained by `MCMC_BiMarkers` and SnappNet on the networks of Figure 1 (in one case we give all possible likelihoods). We have added one sentence acknowledging this in the manuscript (line 316).

2. The language in the introduction is a little oversimplified, especially the sentence "Mechanisms at stake lead a genome to have different parent species - in contrast with the simpler image that depicts a genome as being vertically inherited with modifications from a single ancestral genome." Under the multispecies coalescent a species can have a single parent, but inherited multiple ancestral genomes at the top of the branch. This is followed an overview of different kinds of "mechanisms leading to mix genome contents" (grammatically incorrect, maybe this should be "mechanisms leading to mixed inheritance"?), including HGT in prokaryotes. I think it would be better to remove this or make it clear that the model implemented here isn't useful to model HGT in prokaryotes, since it relies on the free recombination between sites facilitated by sexual reproduction.

   • There was indeed some potential confusion here. We completely re-wrote this part (starting at line 22). The new text also makes it clear that we do not model HGT in this paper.

3. A limitation inherent to inference of species networks using biallelic markers is the apparent non-identifiability of reticulation node heights (between upper and lower bounds). This is shown in section 2.1 of Cao, Zhen, et al. "Practical aspects of phylogenetic network analysis using phylonet." bioRxiv (2019). The authors may consider including this in the discussion, as it seemingly the case that precise inference of reticulation node heights requires other kinds of methods (e.g. multilocus methods or coalescent HMMs). However this does not diminish the utility of Rabier et al.'s method for the inference of the species network topology or speciation times.

   • We thank the reviewer for pointing this interesting paper to us. Section 2.1 in that paper shows that if only one individual is sampled below a reticulation node, then the height of that reticulation node is non-identifiable, and that identifiability is recovered when more than one

individual is sampled. This was also observed by Zhu and Degnan (Systematic Biology, 2017). We now cite both papers at the end of the Introduction, and we discuss the interesting topic of identifiability in more detail in the Discussion (starting at line 920). We are not aware of any identifiability issue that is specific to biallelic marker data.

I enjoyed reading the manuscript and am looking forward to seeing the method used in practice.

- We thank the reviewer for the positive comment.

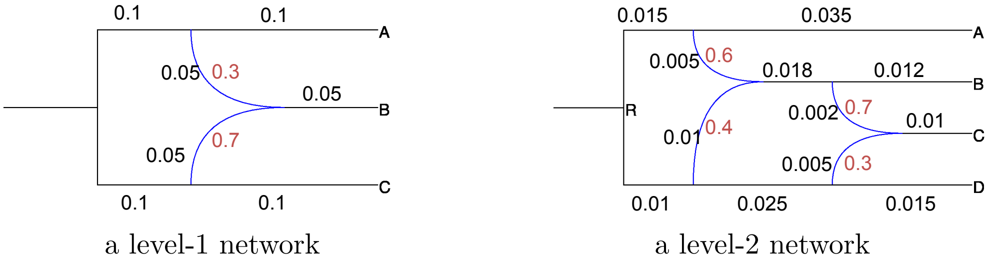

Fig. 1: Two networks used to compare the likelihoods obtained by `MCMC_BiMarkers` and SnappNet. The inheritance probabilities are in red, and the branch lengths are in black. We considered $\theta = 0.005$ for all branches and we set $u = 1$ and $v = 1$.

Tab. 1: All possible likelihoods obtained by `MCMC_BiMarkers` and SnappNet for the level-1 network of Fig. 1. The numbers of lineages were set to $n_A = 2$, $n_B = 3$, and $n_C = 2$.

(r_A, r_B, r_C)	`MCMC_BiMarkers`	**SnappNet**
$(0,0,0)$	0.3158133718700552	0.31581337186422315
$(0,0,1)$	$1.8536603716935 \times 10^{-3}$	$1.853660371657668 \times 10^{-3}$
$(0,0,2)$	0.056772362839906186	0.05677236283895234
$(0,1,0)$	$1.6755618679517247 \times 10^{-3}$	$1.6755618678903335 \times 10^{-3}$
$(0,1,1)$	$1.0719114102957123 \times 10^{-5}$	$1.0705941050619642 \times 10^{-5}$
$(0,1,2)$	$4.7898006674265697 \times 10^{-4}$	$4.789800667080107 \times 10^{-4}$
$(0,2,0)$	$9.884301576846214 \times 10^{-4}$	$9.884301576368968 \times 10^{-4}$
$(0,2,1)$	$7.570444581928021 \times 10^{-6}$	$7.570444581342921 \times 10^{-6}$
$(0,2,2)$	$5.332292032829108 \times 10^{-4}$	$5.3322920321306 \times 10^{-4}$
$(0,3,0)$	0.04027355605294687	0.04027355605172049
$(0,3,1)$	$5.910438937586813 \times 10^{-4}$	$5.910438937463977 \times 10^{-4}$
$(0,3,2)$	0.07852626659254809	0.07852626659131974
$(1,0,0)$	$1.9618887485701787 \times 10^{-3}$	$1.9618887485350735 \times 10^{-3}$
$(1,0,1)$	$1.216270778830845 \times 10^{-5}$	$1.2162707780799976 \times 10^{-5}$
$(1,0,2)$	$4.8281551687985884 \times 10^{-4}$	$4.828155168689878 \times 10^{-4}$
$(1,1,0)$	$1.0989010304649967 \times 10^{-5}$	$1.0989010303396982 \times 10^{-5}$
$(1,1,1)$	$9.099282927803424 \times 10^{-8}$	$9.099282927274783 \times 10^{-8}$
$(1,1,2)$	$7.300548380234911 \times 10^{-6}$	$7.300548379825278 \times 10^{-6}$
$(1,2,0)$	$7.300548380235193 \times 10^{-6}$	$7.30054837982544 \times 10^{-6}$
$(1,2,1)$	$9.099282927803262 \times 10^{-9}$	$9.099282927274914 \times 10^{-9}$
$(1,2,2)$	$1.0989010304649364 \times 10^{-5}$	$1.098901030399711 \times 10^{-5}$
$(1,3,0)$	$4.8281551687985477 \times 10^{-4}$	$4.821551686898895 \times 10^{-4}$
$(1,3,1)$	$1.2162707788308018 \times 10^{-5}$	$1.2162707788079851 \times 10^{-5}$
$(1,3,2)$	$1.961888748570057 \times 10^{-3}$	$1.9618887485350622 \times 10^{-3}$
$(2,0,0)$	0.07852626659255069	0.0785262665913196
$(2,0,1)$	$5.91043893758687 \times 10^{-4}$	$5.910438937463979 \times 10^{-4}$
$(2,0,2)$	0.040273556052945414	0.040273556051720324
$(2,1,0)$	$5.332292032829185 \times 10^{-4}$	$5.332292032130451 \times 10^{-4}$
$(2,1,1)$	$7.570444581927754 \times 10^{-6}$	$7.5704445813427665 \times 10^{-6}$
$(2,1,2)$	$9.884301576845596 \times 10^{-4}$	$9.884301576368857 \times 10^{-4}$
$(2,2,0)$	$4.7898006674265193 \times 10^{-4}$	$4.789800667080225 \times 10^{-4}$
$(2,2,1)$	$1.0719114102956533 \times 10^{-5}$	$1.0719114102479618 \times 10^{-5}$
$(2,2,2)$	$1.6755618679516104 \times 10^{-3}$	$1.6755618678903448 \times 10^{-3}$
$(2,3,0)$	0.05677236283990392	0.0567723862838952165
$(2,3,1)$	$1.8536603716912297 \times 10^{-3}$	$1.8536603716576576548 \times 10^{-3}$
$(2,3,2)$	0.31581337187003317	0.31581337186422315

Tab. 2: A few representative likelihoods obtained by `MCMC_BiMarkers` and SnappNet for the level-2 network of Fig. 1. The numbers of lineages were set to $n_A = 3$, $n_B = 3$, $n_C = 3$ and $n_D = 3$.

(r_A, r_B, r_C, r_D)	`MCMC_BiMarkers`	**SnappNet**
$(0,0,0,0)$	0.420388330654925	0.420388330446373
$(0,0,0,1)$	$2.14133916774914 \times 10^{-3}$	$2.1413391677020254 \times 10^{-3}$
$(0,0,0,2)$	$1.1379876974516707 \times 10^{-3}$	$1.1379876974211235 \times 10^{-3}$
$(0,0,0,3)$	0.0180443910654555995	0.018044391063547768
$(1,0,1,1)$	$5.743150539369824 \times 10^{-8}$	$5.7431505391794586 \times 10^{-8}$
$(1,1,1,1)$	$3.19077116513984 \times 10^{-10}$	$3.1907711650231237 \times 10^{-10}$
$(1,2,1,1)$	$2.0749049389306833 \times 10^{-10}$	$2.0749049388423288 \times 10^{-10}$
$(1,3,1,1)$	$1.2685272071656176 \times 10^{-8}$	$1.268527207088679 \times 10^{-8}$
$(1,3,2,1)$	$2.0091067873781274 \times 10^{-8}$	$2.0091067872430637 \times 10^{-8}$
$(1,3,3,1)$	$3.27797341933502 \times 10^{-6}$	$3.277973419215861 \times 10^{-6}$
$(3,3,3,3)$	0.42038833065492026	0.420388330646373

Review 3

The authors developed a new Bayesian method, SnappNet for phylogenetic network inference. The authors use a faster new method based on joint conditional probabilities to compute likelihoods of phylogenetic networks resulting in faster analysis by orders of magnitude and with an order of magnitude less memory usage than the recent MCMCBiMarkers method for inferring networks.

This work was inspired by the authors of MCMCBiMarkers noting that it is necessary to improve the speed of such methods. The authors leverage the improved speed to consider more complex evolutionary scenarios with an example of inferring a new evolutionary scenario on rice genomic data that is compatible with available evidence.

This is an important development and will be of great interest to the readers of PLOS Computational Biology. The new fast likelihood computation method for networks is novel and useful. However, the analysis of the inferred networks needs major revisions. The treatment of the complicated topic of convergence in a Bayesian analysis needs to be substantially expanded. The authors seem to consider the inferred posterior probability of a network as an accuracy measure and there are major questions about whether the method is correctly inferring the expected network or simply stuck at that network. Finally, The paper needs a much more careful analysis of the differences between SnappNet and MCMCBiMarkers results in order to support their accuracy claims.

Specific comments follow

1. Page 12 - MCMC Operators

   This section is woefully short considering the great importance of MCMC operators on the convergence and accuracy of a Bayesian analysis. What to the most important operators do? How are the operators tailored to work with a network as opposed to a tree? Which operators are most used and what are the acceptance rates? Is there any possibility that the operators have a bias towards or away from certain types of network shapes? If these questions have not been answered in specific detail yet then they should be included in the discussion and mentioned as important future work. I see that the authors cite SpeciesNetwork as the origin of the network topology operators but it is important to mention such concerns at the very least, particularly given that you are combining them with new parameters and new operators.

   • There was indeed too little information and discussion about MCMC operators. We now describe them in more detail in the section "MCMC Operators", which is now significantly longer than in the original version (cf. line 359 onwards).

   • Moreover, as suggested by the reviewer, the questions above about operators and their efficiency are now mentioned as important future work in the Discussion (new paragraph starting at line 896).

   • Finally, note that operators acceptance rates are given at the end of every SnappNet run, since SnappNet is built on Beast. So it easy for any user to inspect them. As an example, in the new Section 8.2.4 of the Supplementary Material (part of a larger set of new experiments), we provide the mean operator acceptance rates across several MCMC runs for data simulated using network B.

2. Page 13 - Phylogenetic networks studied

   Why did you study these three networks? Are they representative of important type of networks or biologically important in some way? Are there any caveats to focusing on these networks or areas of future work that should be studied on larger or more complex networks?

• We rewrote the paragraph starting at line 402, where we now explain that networks A and B allow us to compare SnappNet to `MCMC_BiMarkers` without having to rerun the latter. Network C allows us to study the influence of nested reticulations on the inference. We also clarified (line 411) that networks C(3) and C(4) were chosen because nested reticulations represent a computational challenge for inference tools, and were thus used to test computational efficiency.

• Moreover, we agree that experimenting over more complex and larger networks will be important. We have added a new paragraph about this to the Discussion (starting at line 909), where we also discuss the challenges that this will involve.

3. Page 13 - Bayesian Analysis

   I see in the commands in the supplemental material that you sampled every 1000 iterations of the MCMC. This is worth mentioning in the paper proper along with an explanation that this is to prevent autocorrelation and part of the reason that computational complexity of likelihood calculations is important.

   • Indeed this was missing. It is now explained in the new text starting at line 448.

4. Why did you use a single starting tree or network for each MCMC analysis instead of a random starting point? This is irregular in MCMC analysis and makes it difficult to generalize your results. This needs more explanation and justification.

   • SnappNet is built on Snapp and SpeciesNetwork, and both these packages use a single starting tree as starting point in the default settings. We did compare the results obtained with the single fixed starting tree with those obtained starting from other trees, as well as random networks generated from the prior distribution (cf. the new experiments in sec. 8.2 of the Supplement). The starting point had limited influence on the results in these tests.

   • As an example, if we compare the results for the following starting points (the first is the same as in the manuscript):
   `(((C:0.05,R:0.05):0.05,((A:0.05,L:0.05):0.025,Q:0.075):0.025):0);`
   `(((A:0.05,Q:0.05):0.05,((C:0.05,L:0.05):0.025,R:0.075):0.025):0);`
   `((((C:0.05,A:0.05):0.05,((R:0.05,Q:0.05):0.025,L:0.075):0.025):0);`
   then, on the data sets with 10 000 sites and 1 lineage for species, the average posterior probability of network A was 100% for all 3 starting trees, whereas that of network B was 81.25%, 88.24% and 92.79%, respectively. Note that the reported 81.25% is the lowest for SnappNet among the 3 runs.

   • We made sure that the issue of the initial MCMC state is now more clearly explained in the manuscript (cf. the new text starting at line 419), and — since we now agree that random initial networks are a better choice — we made sure that the new experiments we report in the Supplement (Sec. 8.2) start from an initial state randomly drawn from the prior distribution. For the new rice data sets, we compared the results obtained for different starting points and obtained matching posterior distributions.

5. Page 14 - MCMC convergence

   More detail is needed on what parameters the ESS was measured on. The linked BEAST tutorial mentions that no topological ESS measures are included by default so I am not sure what ESS was measured and whether any tests of topological convergence were performed.

• Indeed this was unclear. We have significantly expanded this section (starting at line 439) to clarify all this. No topological ESS measure was indeed used and topological convergence was only assessed by looking at the similarity between the results obtained for different MCMC replicates. The need for methods to evaluate the autocorrelation and/or ESS of the sampled network topologies is now described explicitly in the Discussion (new paragraph starting at line 879). See also our answer to the next point.

6. In general the explanation of convergence needs to be expanded. Convergence is a complicated yet important topic and a threshold of ESS is most certainly not enough by itself to assume convergence, that is an ESS of each important parameter is a necessary but not sufficient requirement to assume convergence. Importantly, convergence of treelength or network length does not imply the convergence of topology. ESS measures for tree topology have been recently developed and the authors should note whether they have considered similar measures for network topology ESS or indicate that this is an important area for future work. If a topology ESS is not used (or possible) then at the very least multiple MCMC replicates should be tested and the results compared to determine if similar topologies, split frequencies, or network edges are explored before assuming convergence.

   • Aside from clarifying this point in the Methods section (line 439 onwards), this is now acknowledged and discussed in a new paragraph starting at line 879. In that text, we also explain that the main difficulty of adapting existing approaches (such as that of Lanfear et al. for trees) probably lies in the choice of suitable distance metrics for network topologies.

   • Also note that whenever we looked at multiple MCMC replicates on the same dataset, we observed very similar posterior distributions. We only noticed important differences when changing the prior distribution (and even then, the topology was rarely affected). See for example the new results we report in Sec. 8.2 of the Supplement.

   • Finally and very importantly (related to point 8 below), please remember that in all our experiments on simulated data, we deal with extremely small phylogenies (5 taxa!). We believe that **with 10,000 or more sites, the posterior distribution will often be entirely concentrated in a single network topology**. (We certainly expect this to be true for 5-taxon trees estimated from alignments of length 10,000 or more, while for networks this probably depends on their complexity.) For example, the concentration of the posterior in a single network topology was also observed for $> 10,000$ sites by Zhu et al. (see top panels in Figure 5 of that paper). In these cases, once convergence is reached, the MCMC will spend about 100% of the time sampling from a single topology. All topological plots that could be produced in these cases (e.g. topological distance from a focal phylogeny) would consist of a horizontal straight line. The definition of autocorrelation time and ESS for these kinds of "constant" samples is either problematic or not helpful (in case it diagnoses failed convergence when convergence has been in fact reached).

7. page 15 - Study of networks A and B. Table 1 should be closer to its usage.

   • The positioning of Table 1 and of several figures have been improved.

8. page 15-16 Ability to recover the network topology

   I am very confused about what your measure of accuracy is supposed to be for the correct network topology here and in Table 1. From the text it seems that you are reporting the

percentage of total topologies sampled that matched the target/starting topology. However, in a Bayesian MCMC analysis the percentage of samples should be proportional to the posterior probability of that topology, not some measure of accuracy. We should expect the true network to be the most frequently sampled one if the model is correct but values approaching 100% do not seem worth applauding as they may indicate lackluster topology proposals and that the analysis is stuck at the initial network, or alternatively that the analysis is relatively trivial.

• Please note that our analyses do not use the correct network topology as starting point of the MCMC (nor a tree displayed by the correct network). Thus, we do not think that reaching 100% is a symptom of getting stuck at the initial network. Instead, we believe that the reviewer correctly suspected that the analysis is often **statistically trivial**: when the number of sites is in the tens of thousands, the simulated data causes the posterior to be concentrated in a single network topology. This is definitely true for network A, as independent evidence for this can be found in the results shown by Zhu et al. in the paper on `MCMC_BiMarkers`: for 10,000 sites or more, the topological distance between the sampled networks and network A is always 0! (Note the absence of error bars in the pink plots of Fig. 5 in that paper.) Note however that the analysis is definitely **not computationally trivial**, even for these simple networks.

• Nevertheless we completely agree with the reviewer that one cannot equate the posterior probability of the correct topology to the accuracy of a particular method. Indeed posterior probabilities depend on the data, model and priors chosen, not on the algorithmic method. For this reason, we removed most occurrences of the word "accuracy" in the Results section (we only left that word when it referred to the ability to precisely estimate continuous parameters). We wrote "average posterior probability of the correct topology" whenever it applied. We also clarified the way this is computed by rewriting the text starting at line 457.

9. Similarly, the accuracy on network B has not "decreased slightly to 81.25%" but rather the posterior probability of network B is 81.25% indicating that other scenarios are possible.

   • Like for several other similar claims, we have rewritten this sentence (line 515).

10. A better analysis should probably be based on some idea of consensus networks, as an analog to consensus trees, and whether the MCMC analysis can efficiently sample from the probability space and recover that consensus. Some form of consensus analysis could be used to determine the posterior probability of individual splits or network edges and I suspect, as with trees, this will be necessary for studying larger or more complex scenarios where the true network may have a relatively small probability.

    • These are indeed interesting and important ideas. Methods to inspect posterior probabilities of individual splits, and to construct consensus networks are important future developments. These ideas are now discussed in a new paragraph starting at line 909, where we also mention the algorithmic challenges that they may entail.

11. Page 18 - It's difficult to justify the conclusion that MCMCBiMarkers is not performing well if MCMCBiMarkers was not run to convergence. Testing with larger ESS values is not the same as running it until the MCMC has fully converged.

• Note that we actually did not use the ESS values here. As we say at line 626, we let `MCMC_BiMarkers` run for a fixed number of iterations instead of adopting an ESS criterion. Recall that we first ran `MCMC_BiMarkers` for 1.5 million iterations (consistent with Zhu et al.), and then for 12 million iterations (on a subset of cases). This involved very long running times (and CO_2 emissions!). For example, one of the runs with 4 lineages and 1.5 million iterations took us 4 months of wall-clock time. For these reasons, we believe that we have done our best to not misrepresent the performance of `MCMC_BiMarkers` on these data. Note that SnappNet ran for a maximum 804,000 iterations for 10,000 sites, and a maximum of 555,000 iterations for 100,000 sites.

• Also note that we have largely changed the conclusions we draw from these results (paragraph starting at line 642). We now avoid favoring any particular explanation for the fact that `MCMC_BiMarkers` assigns low posterior probability to network C. Namely we are very careful to avoid implying that `MCMC_BiMarkers` has fully converged even when run for 12 millions iterations.

12. Page 22 - You mention here that on the real datasets with free exploration of the topology space the analysis frequently got stuck in local optima. As with the simulated data this suggests that new methods are needed to sample from networks.

• More work on MCMC over networks is definitely important in the future (see the new additions to the Discussion). As for our analyses on rice, we were indeed quite unsatisfied with the difficulty of reaching convergence. Because of this, at the time of the first submission (October 2020) we launched MCMC analyses of 3 new data sets, whose main difference with the old data sets is that they include 3 new taxa representing 3 sub-populations of wild rice.

• The increased taxon sampling — combined with the fact that some of these analyses have been running uninterrupted until now (about 7 months) — allowed us to reach results that we now consider satisfying, especially because they converged to topologically identical results (except in one case). This new Bayesian analysis is now described and discussed in new text appearing at lines 470, 687, and 848.

13. You explain here that you focused on direct analysis of 10 specific topologies by computing their likelihoods and using AIC and BIC penalties. This is one solution but it needs to be more carefully explained to the reader what this means. You should also explain carefully here that this is not a Bayesian analysis as you have moved from posterior probability to likelihood comparisons. The networks can be compared against each other but it is not possible to consider their probability with this type of analysis and it is possible that some other network you do not consider or specific network features have a higher probability than any of these 10 networks.

• Given that we now obtained satisfying results for the Bayesian analysis of the rice data, the likelihood analysis of 10 specific topologies has now been removed. It was definitely less appealing than a fully Bayesian analysis not focusing on any specific network topology.

14. Page 27 - the analysis of the candidate networks is interesting in that it discusses biologically relevant factors and the penalized likelihood ranking of the networks but I think some of the conclusions here are too strong based on just this evidence, such as suggesting that specific networks should be studied more closely based on this analysis or that specific networks should be discounted because their results would be surprising biologically.

• The rice specialists among us have rewritten our conclusions in the light of the new results. Our networks are consistent with previous studies.

15. The mention of heated chains for future work seems out of place here in the results section and should likely be in the discussion or conclusion.

    • We agree. We moved this sentence to the Discussion section.

16. Page 29 - the comparison of SnappNet and MCMCBiMarkers ”accuracy” on network C again seems to be without context. More justification is needed to prove that the low accuracy of the expected network is a detriment. For example, I suggest discussing the posterior probability from each method that the network has a given number of reticulations to better make your claim that MCMCBiMarkers is discounting these scenarios.

    • In the answer to comment 11, we have already commented on the fact that the conclusions drawn from these results have been softened.
